# Bioinspired gelatin based sticky hydrogel for diverse surfaces in burn wound care

**DOI:** 10.1038/s41598-022-17054-w

**Published:** 2022-08-12

**Authors:** Benu George, Nitish Bhatia, Abhitinder Kumar, Gnanamani A., Thilagam R., Shanuja S. K., Kannan Vadakkadath Meethal, Shiji T. M., Suchithra T. V.

**Affiliations:** 1 School of Biotechnology, National Institute of Technology Calicut, Kozhikode, India; 2 Department of Pharmacology, Khalsa College of Pharmacy, Amritsar, Punjab India; 3 School of Medical and Allied Sciences, G. D. Goenka University, Haryana, India; 4 CSIR-Central Leather Research Institute, Adyar, Chennai, India; 5 Division of Biochemistry and Molecular Biology, Department of Zoology, University of Calicut, Kozhikode, India

**Keywords:** Biomaterials, Preclinical research, Biomaterials, Biomimetics, Tissue engineering

## Abstract

Proper burn wound management considers patient’s compliance and provides an environment to accelerate wound closure. Sticky hydrogels are conducive to wound management. They can act as a preventive infection patch with controlled drug delivery and diverse surface adherence. A hypothesis-driven investigation explores a bioinspired polydopamine property in a gelatin-based hydrogel (GbH) where polyvinyl alcohol and starch function as hydrogel backbone. The GbH displayed promising physical properties with O–H group rich surface. The GbH was sticky onto dry surfaces (glass, plastic and aluminium) and wet surfaces (pork and chicken). The GbH demonstrated mathematical kinetics for a transdermal formulation, and the in vitro and in vivo toxicity of the GbH on test models confirmed the models’ healthy growth and biocompatibility. The quercetin-loaded GbH showed 45–50% wound contraction on day 4 for second-degree burn wounds in rat models that were equivalent to the silver sulfadiazine treatment group. The estimates for tensile strength, biochemicals, connective tissue markers and NF-κB were restored on day 21 in the GbH treated healed wounds to imitate the normal level of the skin. The bioinspired GbH promotes efficient wound healing of second-degree burn wounds in rat models, indicating its pre-clinical applicability.

## Introduction

Humankind’s quest for inspiration from nature has led to the successful creation of novel and functional hydrogels^1^. The biomimicking of slug-based protective mucus contributed to the development of tough, surface-sticking hydrogels of an alginate-polyacrylamide combination^2,3^. An effective dopamine complex in surface adhesive hydrogel design, inspired by aquatic animals, such as mussels, where dopamine acts as the main ingredient for underwater adhesion, has been recently developed^4^. Many bioinspired hydrogels have been designed using the biosystem as a model for understanding its diverse functions, from its molecular architecture to its macroscopic geometry^5^. Hydrogels are complex three-dimensional (3-D) networks of hydrophilic polymer chains and given their hydrophilic nature, they contain significant quantities of water^6,7^. They exhibit swelling when exposed to water, especially since the human body has water as a major component and hydrogels can contain high volumes of water. Thus, allowing them to be an excellent candidate for various biomedical uses, like tissue engineering, drug delivery, self-healing materials, biosensors, and hemostatic bandages^8,9^.

The largest and essential organ of our body, the skin, is an outer defensive layer. Classical wound dressing materials such as dry fabrics (absorbent gauze or cotton), have minimal medicinal benefits, involve pain, and require frequent adjustments in dressing, thus causing the patient continued distress. Hydrogels are promising as they promote healing by maintaining a proper humidity level at the wound site. Most wound care studies consider hydrogels the best candidate for wound dressings, as they have a 3-D structure that resembles the natural extracellular matrix, which guarantees the wound a humid atmosphere^10,11^. The epithelial fractures and connective systems underpin the human body’s ability to ensure sufficient protection from external harm^12^. The skin seems to be the most vulnerable of all human body organs, from bruises and scratches to burns. Statistically, burn injuries are the fourth most frequently encountered debilitating form of trauma^13^. An ideal burn wound dressing is expected to promote recovery in shorter periods and relieve pain since burn wounds call for prolonged medical attention.

In the last decade, mussel-inspired catechol chemistry has become an intriguing part of science, especially in hydrogels^14^, where polyacrylamide and bis-acrylamide compositions are a common matrix for a catechol-trapped hydrogel system^15^. Research indicates that prolonged or frequent contact of polyacrylamide and bis-acrylamide with the skin can trigger dermatitis and cancer in animal models^16^. Pre-clinical findings suggest that continued exposure to polyacrylamide and bis-acrylamide compositions in animal model studies compromised reproductive and nervous systems^17^. Even though multiple dressings are readily available on the market, an innovative wound treatment solution must be established to deal with burn injuries. The current investigation is predicated on the hypothesis that ‘polydopamine does exhibit an adhesive property in a non-toxic composition of hydrogel formulation’. Hence, the GbH wound dressing was developed and its physical and biological performance on diverse surfaces was evaluated. Finally, an evaluation of the drug release pattern from the wound dressing hydrogel patch was assessed to understand the formulation’s drug diffusion pattern in wound healing of the second-degree partial burns in rat models.

## Results

### Synthesis and optimization of functional GbH

**Figure 1:**
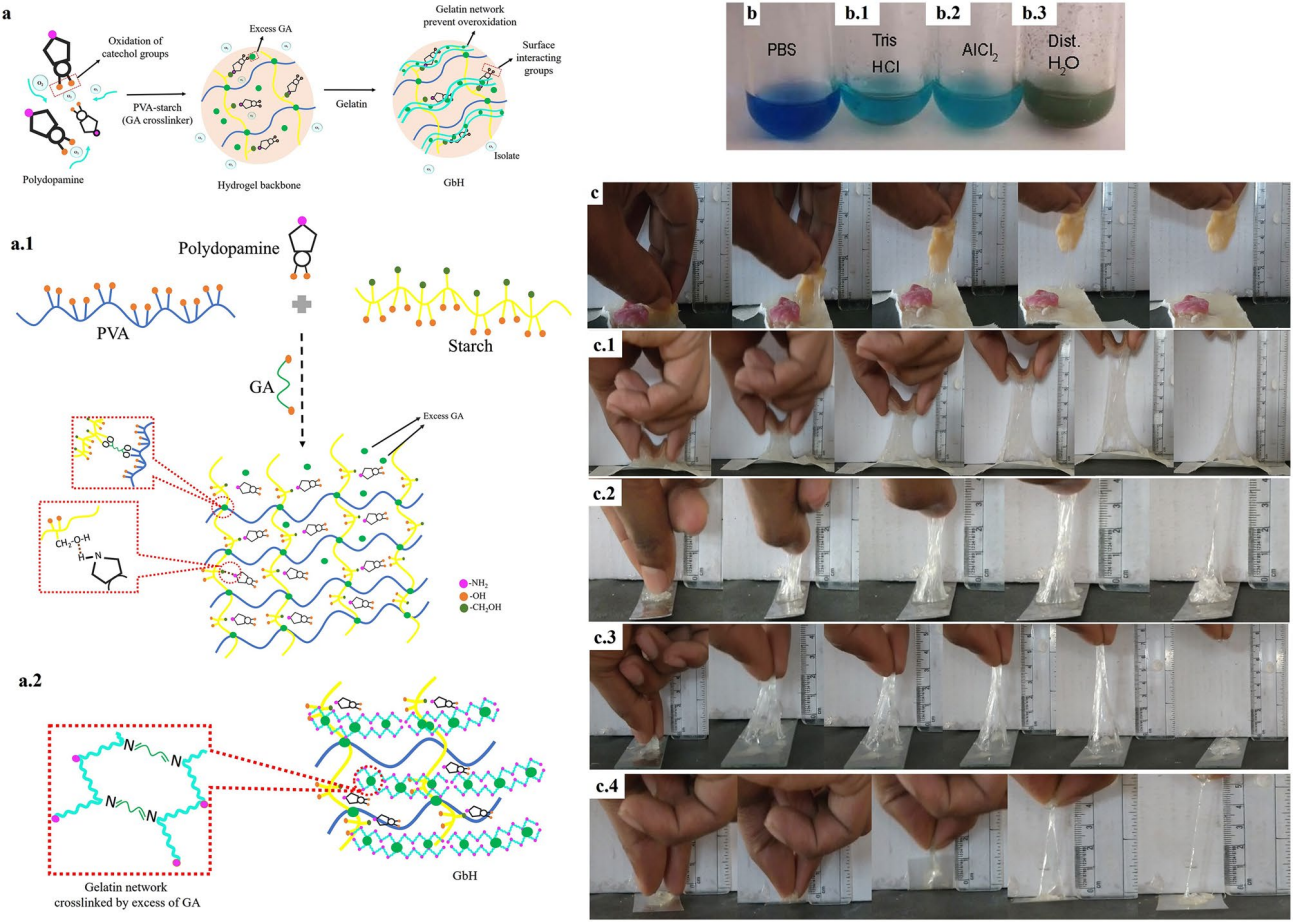
Schematic and diverse performance of GbH: (**a**) Schematic of the developed diverse surface gelatin-based hydrogel (GbH); (**a.1**) The developed GbH is a two-stage process where polymers of polyvinyl alcohol (PVA) and starch, and the optimized alkali polymerized polydopamine concentration is chemically crosslinked with glutaraldehyde reagent (GA), which act as the base gel; (**a.2**) The excess glutaraldehyde in the semi-polymerized stage crosslinks with gelatin and forms a polymer network over the base gel. The gelatin network prevents external oxygen and inhibits the oxidation of catechol groups.; The Benedict test was used to determine excess glutaraldehyde comprised: (**b**) PBS, (**b.1**) Tris–HCl, (**b.2**) AlCl_2_ and (**b.3**) distilled water. The developed GbH with stabilizer on diverse surfaces: (**c**) chicken; (**c.1**) pork; (**c.2**) stainless steel surface; (**c.3**) glass surface and (**c.4**) plastic surface.

The classical optimization adopted in the present research has led to the development of the GbH with a bioinspired polydomine’s sticky property^2,18^. The PVA hydrogels have numerous applications in the pharmaceutical and biomedical industry due to their ease of processing, biocompatibility, non-carcinogenicity, bioadhesiveness, non-toxicity and transparency nature. Blending of synthetic hydrogels such as PVA, poly-caprolactone and poly-lactic acid with starch ensures the abundance of hydroxyl groups and improve the mechanical properties^19–21^. Starch, a biopolymer abundantly available in nature, is cost-effective, widely available with thermoplastic and efficiently biodegradable property. However, a starch-based system frequently exhibits poor mechanical, brittle, and highly water-soluble properties, and a synthetic polymer modification proves to be advantageous^21,22^. In the current study, the PVA-starch hydrogel blend, chemically crosslinked by glutaraldehyde reagent (GA) (Fig. 1a) obtained from the solution casting process was transparent and had no adhesive or sticky property. The blend of PVA-starch hydrogel served as a base to add a sticky property. Various concentrations of polydopamine were added to the hydrogel base and then the elongation test was performed (Tables 1 and 2). The lowest concentration of 0.5% was found to be elastic and appropriately sticky (Fig. 1a.1), as desired of a hydrogel formulation since a wound dressing should withstand any external force as well as protect the wound.

**Table 1:** Physical nature of GbH.

Sl. no	Polydopamine (%)	Characteristics
1	0.5	Elastic and sticky
2	1	Elastic and sticky
3	1.5	Elastic and sticky
4	2	Firm and sticky
5	2.5	Firm and sticky
6	3	Firm and sticky

**Table 2:** Elongation property of ﻿GbH.

Sl. no	Polydopamine (%)	Elongation(mm) (mean ± SD)
1	0.5	105.52 ± 2.13
2	1	142.45 ± 3.12
3	1.5	155.30 ± 5.14
4	2	136.53 ± 4.31
5	2.5	123. 74 ± 2.16
6	3	137.75 ± 4.53

Incorporating gelatin and sodium metaperiodate in the semi-polymerized state ensured the desired sticky property, which persisted for four days at RT (room temperature, 22 ± 3 °C). The best combination was observed as 500 μL of gelatin with a ratio of 1:5 of polydopamine and sodium metaperiodate. Previous research on mussel-inspired polydopamine-polyacrylamide hydrogel maintained sufficient free catechol groups in the hydrogel and prevented overoxidation of polydopamine^4^. The polyacrylamide in the mussel-inspired hydrogel obstructed the external oxygen and inhibited the oxidation of catechol groups by framing a polymer network. Mussels, in nature, prevent the overoxidation of the catechol group by secreting reductive cysteine-rich proteins, thus maintaining strong adhesiveness^4^. In the current research, the gelatin network prevented overoxidation and hindered the immediate oxidation of the catechol group, thus providing a prolonged sticky property to the hydrogel catechol. The base gel is formed by the crosslinked chain of PVA-starch (Fig. 1a.2). The crosslinking agent GA reacts with the adjacent O–H groups of the PVA, forming cyclic glyoxal^23^. The same crosslinking happens between starch and PVA. The strong H-bonding interactions between PVA-starch also aided the network formation. The polydopamine also interacts with the base gel-forming hydrogen bonds with the N–H present in the structure groups. The excess GA crosslinks the gelatin and makes the network stable^24^. The catechol moieties in the polydopamine can undergo various surface interactions in the normal tissue resulting in the formation of interfacial covalent bonds^25^.

The GbH was subjected to a washing process to eliminate excess GA. Thus, a suitable buffer to promote the removal of an excess of the C–H–O functional group was determined by the Benedict test^26^. The change in colour from dark green to crystal blue in the Benedict test is indicative of the C–H–O group being eliminated. The buffers used were Tris-HCl (3 M), aluminium chloride (AlCl_2_) (50 mM), phosphate-buffered saline (PBS) (0.1 M) and distilled water (Fig. 1b–b.3). The Benedict test revealed that PBS is ideal for washing purposes (Fig. 1b) where the washing was performed in three 6 h-cycles, then the buffer was discarded and the gel refilled with fresh PBS for purposes of gel preservation, which can be stored at 4 °C in the PBS or distilled water. The Fourier transform infrared spectroscopy (FT-IR) spectra of the washed gel also supports the complete removal of GA; the IR details are briefly explained in “Characterization of GbH” section. The stickiness of the GbH to the wet surface is enabled by the surface amine groups present on the hydrogel^27,28^. The GbH was kept on diverse surfaces (overnight at RT) and the stickiness of each hydrogel to the surface was observed (Fig. 1c–c.4). The GbH was placed onto different wet surfaces, such as chicken (Fig. 1c) and pork (Fig. 1c.1) and dry surfaces, such as stainless steel (Fig. 1c.2), glass (Fig. 1c.3) and plastic (Fig. 1c.4). The stickiness to metal, glass and pork was stronger than that of poultry meat.

### Physical performance of GbH

The hydrogel synthesized from polyelectrolytes swells more due to the charge repulsion among the polymer chains^29^. Such property as swelling is desirable for controlled drug release^30^. The GbH is designed for wound care as a dressing material and therefore, the present formulation was loaded with three distinct medications, viz. ciprofloxacin (antibacterial drug), 5-flucytosine (antifungal drug), and quercetin (drug promoting wound healing) (Table 3). In the present study, the GbH was loaded with different concentrations of drug (a minimum of 5 mg/mL and a maximum of 20 mg/mL) depending on the saturation and effective concentration of drugs to be diffused. The drugs were solubilized in dimethyl sulfoxide (DMSO) and 200 μL of drugs were loaded with gelatin. A study of a combination of drugs (ciprofloxacin (100 μL) and quercetin (100 μL)) was also carried out since wound healing requires both bacterial protection and wound healing promoting drugs. The GbH without any drugs was included as a control for comparable properties.

**Table 3:** Elongation property of the GbH.

Drugs	Drug conc. (mg)	Elongation (mm) (mean ± SD)
Ciprofloxacin	5	122.93 ± 3.15
	20	91.91 ± 4.67
Quercetin	5	72.19 ± 2.61
	20	51.84 ± 3.59
5-flucytosine	5	80.52 ± 1.03
	20	75.43 ± 3.77
Ciprofloxacin + Quercetin	5	129.07 ± 3.45
5-flucytosine + Quercetin	5	119.03 ± 2.45
Control	–	126.34 ± 2.32

**Figure 2:**
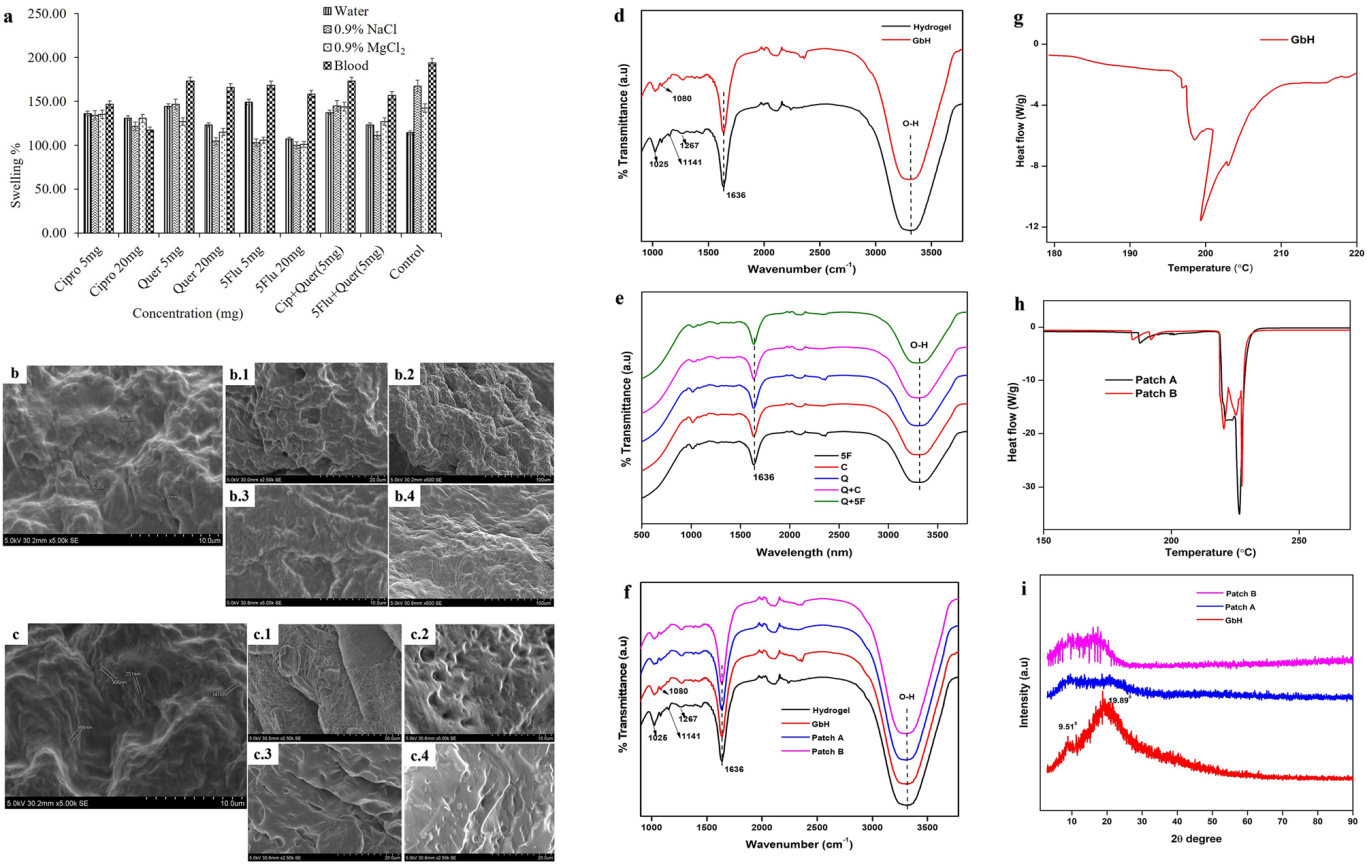
Characterization of the GbH: The physical evaluation of GbH: (**a**) the swelling behaviour of GbH in water, NaCl solution, MgCl_2_ solution and blood. The SEM image: (**b**) and (**c**) control GbH with pore size range from 266-393 nm; (**b.1**) antibacterial ciprofloxacin drug-loaded GbH; (**b.2**) wound healing promoting quercetin drug-loaded GbH; (**b.3**) antifungal drug 5-flucytosine loaded GbH; (**b.4**) Combination of antibacterial drug ciprofloxacin and quercetin loaded GbH. (**c.1**) SEM image of patch-A GbH before the release of salicylic acid; (**c.2**) SEM image of patch-A GbH after the release of salicylic acid (**c.3**) SEM image of patch-B GbH before the release of salicylic acid; (**c.4**) SEM image of patch-B GbH after the release of salicylic acid. Surface functional characteristics illustrated: (**d**) FT-IR of standard hydrogel and GbH; (**e**) FT-IR of GbH with antibacterial drug ciprofloxacin (C) loaded, wound healing promoting drug, quercetin loaded (Q), GbH with antifungal drug 5-flucytosine (5F) loaded, GbH with antibacterial drug ciprofloxacin and quercetin combined (Q + C) loaded and GbH with antifungal drug 5-flucytosine and quercetin combined (Q + 5F) loaded; (**f**) FT-IR of patch-A and -B. DSC characterization of patches: (**g**) GbH and (**h**) patch-A and -B. (**i**) XRD of the GbH as patch-A and -B.

The GbH had exhibited good swelling in various solvents and was comparable to the control (Fig. 2a). Besides, the swelling nature facilitates the absorption of exudates and promotes a suitable environment for wound healing. The GbH did not dissolve in any of the test solvents and the swelling percentage of blood-submerged GbH was observed to be more due to blood components^29^. In the current study the swelling capacity was negligibly compromised as the drug concentration increased. Being a candidate for wound dressing, a hydrogel with low water vapour transmission (WVT) (Table 4) value is favoured, as the fluid loss is limited and a sufficient moist atmosphere for the wound healing is maintained^22^. Reports indicate that an average ~ 250 g/m^−2^/d^−1^ loss of water from normal skin is experienced at 35 °C, whereas in a wound, water loss greatly rises to 5000 g/m^−2^/d^−1^, and loss will be based on the nature of the injury^31^. The drug-loaded GbH was found to have a comparatively low WVT and is comparable to the control. The greater the water depletion, the more impossible it is for the wound to heal^32^. Thus, swelling, WVT and moisture retention capacity (MRC) are essential parameters in hydrogels, which foster exudate absorption and limit the water transfer to ensure a condition conducive to wound healing, thus providing appropriate drug diffusion. There was a reasonably strong MRC in the prepared GbH (Table 4) and all MRC values showed a negligible difference from the control (Table 4) (drug unloaded GbH). The findings showed proper crosslinking even after the GbH had been exposed to 4 days of immersion in distilled water. The prepared GbH had a very strong gel fraction (GF) (Table 4) and all GF values were comparable to the control.

**Table 4:** Physical property of the GbH.

Drugs	Drug conc. (mg)	Physical property (mean ± SD)		
		WVT (g/m^−2^ h^−1^)	MRC (%)	GF (%)
Ciprofloxacin	5	10.49 ± 0.57	47.76 ± 2.01	67.93 ± 3.10
	20	10.49 ± 0.55	41.90 ± 2.08	
Quercetin	5	8.42 ± 0.43	41.90 ± 2.10	62.41 ± 3.12
	20	9.72 ± 0.49	40.21 ± 2.08	
5-flucytosine	5	8.75 ± 0.44	47.04 ± 2.13	66.63 ± 3.13
	20	9.25 ± 0.44	44.72 ± 2.17	
Ciprofloxacin + Quercetin	5	7.13 ± 0.36	44.51 ± 2.13	63.84 ± 3.14
5-flucytosine + Quercetin	5	9.02 ± 0.40	43.51 ± 2.11	64.54 ± 3.13
Control	−	6.15 ± 0.31	45.07 ± 2.12	70.01 ± 3.10

### Characterization of GbH

The surface of the GbH was found to be dense in the scanning electron microscope (SEM) imaging and at very high magnification, few pores were visible, ranging from 266 to 393 nm (Fig. 2b, c). A dense surface benefits as a wound dressing since it does not encourage bacteria to infiltrate the injury site and cause infection easily^15^. A similar investigation on PVA-starch formulations found insoluble starch particles on the surface^33^ that were not visible on the GbH surface^29^. The observation of no surface change when compared with the control GbH indicates that the drugs were appropriately (uniformly/ properly) blended with the GbH, ensuring a uniform drug loading process (Fig. 2b.1–4,b.1–4). Even though the GbH is translucent in nature, its colour can be changed based on the drug-loaded. The colour of the GbH was changed to yellow and a milky turbid when quercetin and 5-flucytosine, respectively, were added.

The IR spectra of the hydrogel and GbH (Fig. 2d) displayed a broad peak in the range 3300–3500 cm^−1^, derived from the intermolecular hydrogen-bonded O–H stretching of the components^34^. The peak observed at 1636 cm^−1^ corresponds to N–H vibration of polydopamine (Fig. 2d), which was seen evidently in all the spectra. Since the spectra evidenced no characteristic aldehyde bands, there will not be any excess of glutaraldehyde. There was no reduction in the intensity of O–H bands even after the incorporation of drugs (Fig. 2e), indicating the abundance of hydrophilic O–H groups contributed by its components. Research indicates that the adhesives characteristics of hydrogels are mainly associated with the surface O–H groups of the hydrogel interacting with chemical groups such as hydroxyl, amino, and carboxyl on the surface of tissues^27,28,35^. The peak at 1141 cm^−1^ revealed C–O stretching arising due to the intermolecular hydrogen bonds formed between neighbouring PVA chains^36^. The stretching vibration exhibited by C–O bond in the C–O–C groups of the starch was found at 1025 cm^−1^ in the GbH, while the peaks corresponding to C–O stretching of the gelatin were found at 1080 cm^−1^ and 1030 cm^−1^ in the spectra of GbH (Fig. 2d). The peak arising due to the presence of a secondary amide group got merged with the N–H band of polydopamine at 1636 cm^−1^. The spectra of the GbH retained all its characteristic peaks in the respective region, revealing that no chemical interactions or structural changes occurred upon the incorporation of the drug (Fig. 2e and f).

The thermal behaviour of the GbH was studied with differential scanning calorimetry (DSC) thermogram. The melting endotherm peak for the GbH is observed at 199 °C (Fig. 2g). This can be mainly ascribed to the major component as PVA; however, the T_m_ observed here is lower than the literature reports for pure PVA, mainly because of the interactions between the PVA chains and other components, making it an amorphous nature of the GbH^37^. The GbH is thermally stable up to 200 °C, while the drug incorporated GbH exhibited higher T_m_ at 216 °C, attributed to the physical interaction between the drug and the GbH (Fig. 2h). The crystalline or amorphous characteristic is revealed through X-ray powder diffraction (XRD) with a peak at 2θ = 9.4°, which depicts the semi-crystalline peak of PVA^38^. The presence of starch in all cases was evident due to the presence of a small shoulder peak at 9° (Fig. 2i). The intensity of the peaks reduced upon the incorporation of the drugs. The diminished intensity of the peaks in patch-A and -B reveals the amorphous nature of the GbH.

### Study of controlled drug release

The drug release profile was assessed using a 1% drug dosage of salicylic acid. Two approaches were implemented: patch-A, where the GbH was prepared and dispersed in salicylic acid; patch-B, where the GbH was immersed in 10 mL of salicylic acid solution in acetone until the solution evaporated. The elongation test of the patches indicated that the method of drug integration did not alter the GbH’s elongation (Table 5). The patches displayed elongation equal to control patches, i.e., the GbH without drug.

**Table 5:** Elongation test of the GbH patches.

Hydrogel	Elongation (%) (mean ± SD)
Patch-A	129.57 ± 4.47
Patch-B	105.97 ± 3.19
Control	126.34 ± 4.93

**Figure 3:**
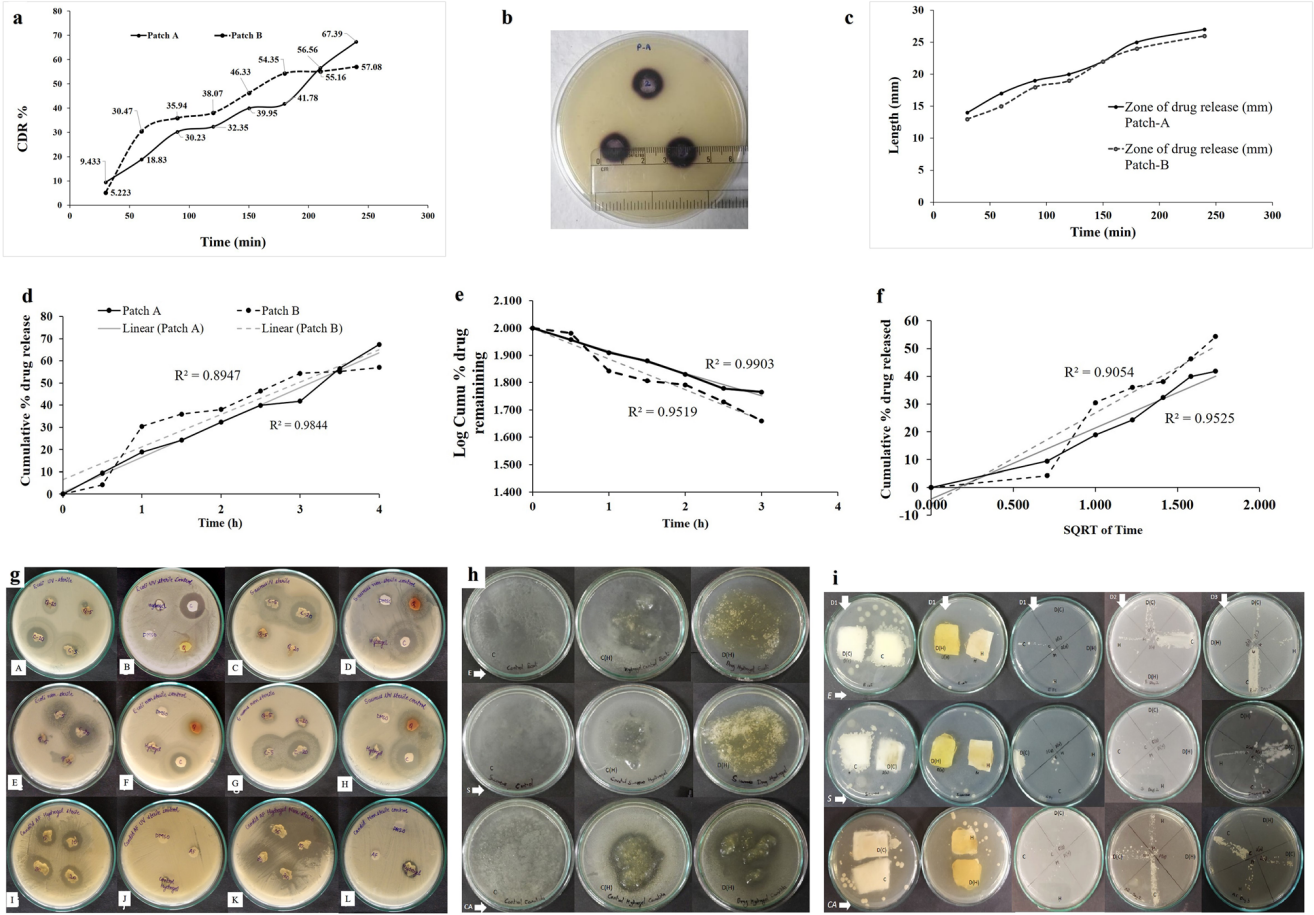
Mathematical modelling for transdermal drug delivery system: Time-dependent evaluation of salicylic acid release from GbH: (**a**) graphical illustration of spectrophotometric analysis of controlled drug release profile of patch-A and -B for salicylic acid; (**b**) illustration of agar well diffusion test for controlled drug release profile of patch-A and -B; (**c**) graphical illustration of controlled drug release profile of patch-A and -B for salicylic acid agar well diffusion test. Mathematical model representation of drug release from GbH: (**d**) zero-order, (**e**) first-order and (**f**) Higuchian release kinetics. Agar diffusion of GbH: (**g.A**) UV-sterile GbH performance against *E.coli;* (**g.B**) Control plate of UV-sterile GbH performance against *E.coli*; (**g.C**) UV-sterile GbH performance against *S. aureus;* (**g.D**) Control plate of UV-sterile GbH performance against *S. aureus*; (**g.E**) Non-sterile GbH performance against *E.coli;* (**g.F**) Control plate of non-sterile GbH performance against *E.coli*; (**g.G**) Non-sterile GbH performance against *S. aureus;* (**g.H**) Control plate of non-sterile GbH performance against *S. aureus*; (**g.I**) UV-sterile GbH performance against *C. albicans*; (**g.J**) Control plate of UV-sterile GbH performance against *C. albicans*; (**g.K**) Non-sterile GbH performance against *C. albicans* and (**g.L**) Control plate of non-sterile GbH performance against *C. albicans*. (**h**) illustrate agar overlay performance of GbH where E, S and CA denote *E. coli, S. aureus* and *C. albicans,* respectively; C denotes Control plate; C(H) denotes positive control GbH; D(H) denotes test drug-loaded GbH. (**i**) illustrates patch agar performance of GbH where D1, D2 and D3 are Day 1, 2 and 3, respectively; E, S and CA denote *E. coli, S. aureus* and *C. albicans,* respectively; C denotes Cotton patch; D(C) denotes drug-loaded cotton patch; H denotes GbH and D(H) denotes drug-loaded GbH.

The sequence of drug release (Fig. 3a) from the patches, represented that both patch-A and -B exhibit a controlled drug release percentage (CDR%) of 60.39 ± 2.25% and 57.08 ± 2.02%, respectively, within a period of 4 h. Patch-A and -B favourably displayed an identical pattern with slow-release; thus, a regulated drug release pattern was observed. A comparable gelatin hydrogel investigation recorded 100% of drug release in 4 h, whereas the GbH exhibited only 60% drug release. Studies suggest that the drug release was faster and the equilibrium achieved was at 3 h in an environment of sufficient dissolution media^39^. Patches-A and -B were observed to have a drug loading capacity of 13.48 ± 0.17 and 11.42 ± 0.15 mg, respectively. The drug loading process showed minor differences in the properties of the GbH, and patch-B showed a partial loss (Table 5) in terms of elongation. The solution casting system is efficient and has a strong potential for drug loading in the present investigation. The agar diffusion drug release investigation produced a violet/purple colour on agar plates due to a complex reaction between diffused salicylic acid and ferric chloride (Fig. 3b,c), where the graph shows the drug release zone vs. time, which determines the drug release kinetics (Fig. 2c). Based on the diameter of the zone formed due to the drug release, the controlled release characteristics of the GbH were observed. The release of salicylic acid was slow and could be observed by the expansion of the ring in a time-dependent manner. The GbH proves to be the ideal formulation for the diffusibility of the drug, which can be explained through further mathematical kinetic observations.

#### Theoretical validation of drug release profile: mathematical kinetics

The CDR% value is further considered for the statistical model analysis of the GbH drug release pattern. The release kinetics in zero- (Fig. 3d), first-order (Fig. 3e), and Higuchian kinetics (Fig. 3f) of the drug release pattern for patch-A and -B were plotted^40^. The graphs reflect the statistical form of the formulation of transdermal drug release. In a zero-order model analysis, data from in vitro drug release trials are plotted as the total amount of drug released vs. time (Fig. 3d). This relationship can explain the drug diffusion of drug release forms, including transdermal systems, matrix tablets with low soluble drugs in coated forms and osmotic systems^41^. The first-order model represents a cumulative log percentage of the drug remaining vs. time, which will result in a straight line with a slope of − K/2303 (Fig. 3e). This association can be used to categorise drug dissolution in prescription dosage types, such as those comprising water-soluble drugs in porous matrices^41^. Higuchian kinetics deals only with the total percentage of drug release vs. the square root of time (Fig. 3f). This correlation can be used to explain the drug dissolution of many kinds of modified release drug dosage formulations, such as transdermal systems and matrix tablets with water-soluble drugs^41^.

The drug release of salicylic acid from the GbH matrix fits the concept of zero- and first-order kinetics (for patch A, r^2^ = 0.984 and r^2^ = 0.99; for patch B, r^2^ = 0.894 and r^2^ = 0.95, respectively) (Fig. 3d,e). Patch-A also follows Higuchian kinetics with a relatively close correlation coefficient (r^2^ = 0.952), but patch-B had a lower value than patch-A (Fig. 3f). Patch-B has a lower value in the zero-order kinetic model compared with patch-A (Fig. 3d). Similarly, A previous report on the gelatin-based hydrogel mathematical models revealed that the hydrogel developed in the same manner as patch-B has a lower drug release than patch-A due to the esterified complexes hindering the salicylic molecule release^39^. In accordance with the validation of the mathematical models for a transdermal drug delivery system**,** patch-A follows three-drug release profiles, i.e., zero-, first-order and Higuchian model, but patch-B had a lower value as compared to patch-A for the zero-order and Higuchian model. A similar drug release pattern has been observed for diclofenac sodium entrapped in polyethylene glycol- and polyethylene glycol-polycaprolactone-based hydrogels^42^. Patch-A and -B, as discussed, obey all three kinetic laws, where the diffusion process releases salicylic acid. The drug uses the same channel used to diffuse it into the matrix, which can be confirmed by SEM analysis before and after the drug release of patch-A (Fig. 2c.1,c.3). The rough surface observed before release is a smooth surface in the after-drug release SEM images (Fig. 2c.2,c.4) of patch-B, which is comparable to the surface of the control GbH (Fig. 2c). The IR results (mentioned in “Characterization of GbH” section and Fig. 2f) showed the prominent presence of O–H functional group on the surface of the GbH and the drug loading did not alter much surface functional groups.

### Drug diffusion performance of GbH

Hydrogel dressings primarily maintain the wound area moist and protect it from infection. The porosity observed in the SEM analysis of the GbH (section: “Characterization of GbH” and Fig. 2b, c) are indicative of advantages like high local concentration of the active ingredient, slow release and swelling. The drug diffusion from the GbH was tested against microorganisms (*Escherichia coli* MTTC 443, *Staphylococcus aureus* MTTC 96 and *Candida albicans* MTTC 183) using agar-based methods such as agar diffusion, agar overlay and patch-agar. These methods showcase drug release efficiency from the GbH and its performance in controlling infections. In the present investigation, the surface-sterilized and -unsterilized GbH (Fig. 3 g.A–g.L) were tested using the agar diffusion method. The GbH demonstrates strong diffusibility in both lower and higher concentrations of drugs, i.e., 5 mg and 20 mg. The observed two distinct rings of the inhibition zone might be due to the slow diffusivity of the drug from the GbH. The sterilized or unsterilized gel has little effect on drug diffusibility via the GbH. The agar overlay system (Fig. 3h) showed strong diffusibility of the drug at lower concentrations (5 mg) and the GbH with test drugs displayed no microbial growth when compared to the controls. The GbH is thus a successful choice for wound protection against bacterial (Fig. 3g.A–g.H) and fungal infection (Fig. 3g.I–g.L).

The tests and controls, as in the patch agar investigation (Fig. 3i), were infected by a swab of gram-negative *E. coli* and gram-positive *S. aureus* for antibacterial studies incubated at 37 °C. Antifungal plates were inoculated with *C. albicans* and incubated at 27 °C. The study was performed over a period of three days where a swab was taken from the contaminated area and monitored for bacterial and fungal growth on nutrient agar and potato dextrose agar plates, respectively. The present findings (Fig. 3i) showed that the GbH loaded with drugs performed superior antimicrobial activity as opposed to the GbH without drugs and controls. A thick microbial growth was observed on the cotton patch drug-loaded sample from day 1. The patch agar system drug-loaded and non-drug loaded proved the GbH’s ability to avoid infection due to its soft wound-dressing bandage with a strong diffusivity property.

### Safety profile of the GbH

#### In vitro cell culture-based toxicity analysis

**Figure 4:**
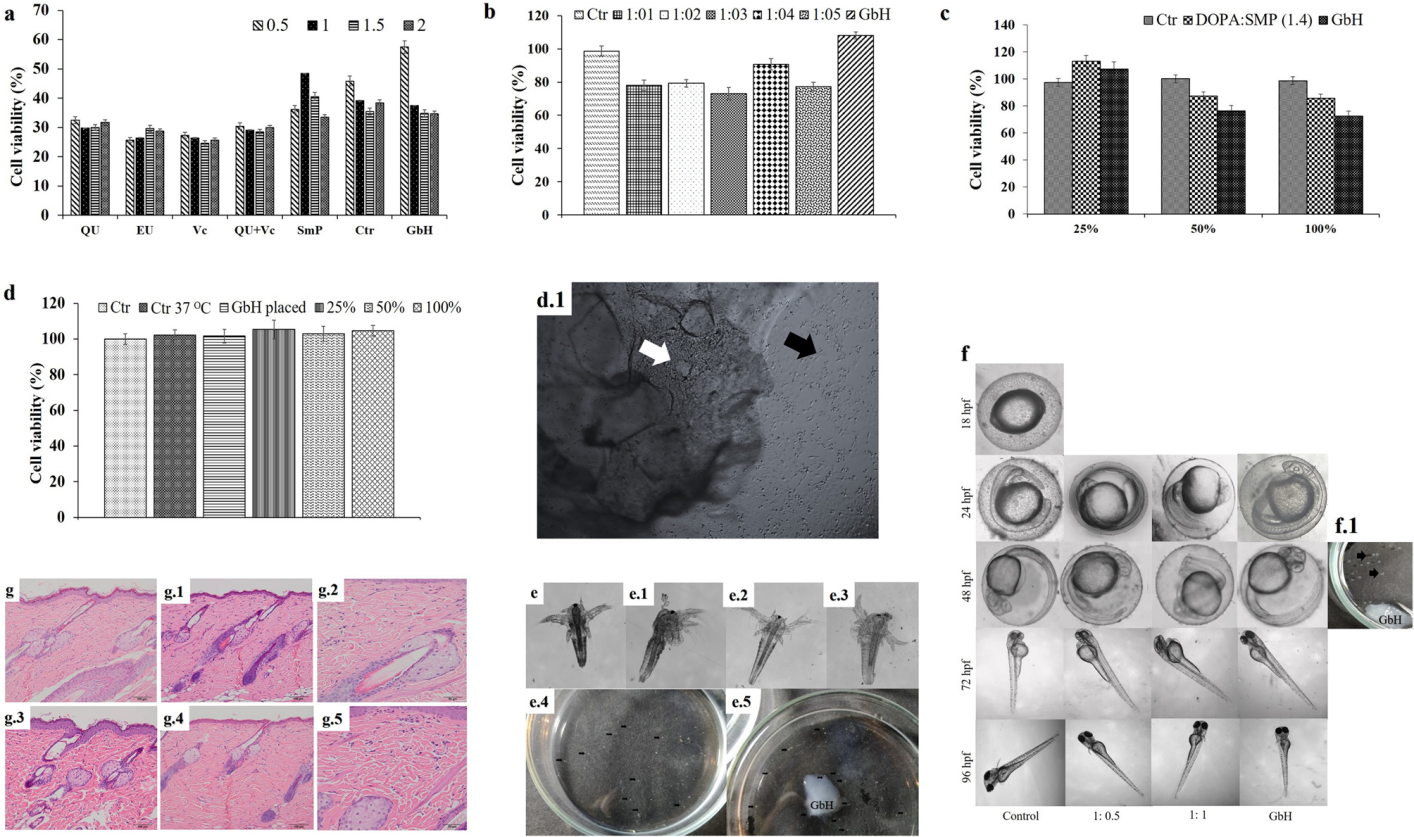
In vitro and in vivo performance of GbH as a dressing material for second-degree burn wounds: Cytotoxicity assessment of the GbH using L929 cells: (**a**) Cell viability of L929 when exposed to GbH for 24 h, where Hb is hydrogel base with no stabilizer and the legend denotes the concentration (mg) of each test stabilizer; (**b**) Cell viability of 3T6 when exposed to the GbH leachate medium for 24 h, where Ctr is the GbH base with no stabilizer and the legend denotes the ratio of polydopamine to sodium metaperiodate and (**c**) Cell viability of 3T6 when exposed to the GbH leachate media for 24 h at a different percentage, where Ctr is the GbH base with no stabilizer and the legend of the ratio denotes polydopamine and sodium metaperiodate ratio in the GbH. (**d**) Cell viability of the HaCat when the GbH is in contact with the cells (GbH placed) and leachate medium at 100% medium replacement incubated for 24 h, where Ctr denotes control, Ctr 37 °C denotes the positive control; (**d.1**) Observed event of healthy HaCat cells after 24 h in contact with the GbH, where white arrow denotes the GbH and black arrow denotes healthy HaCat cells in 96-well plate. Observed development of brine shrimp when exposed to the GbH for 24 h, (**e**) Positive control: nauplii from the aerated tank; (**e.1**) Control: nauplii in artificial saltwater; (**e.2**) and (**e.4**) Test: nauplii in 1:1 medium of the GbH leeched buffer and artificial saltwater; (**e.3**) and (**e.5**) Test: The GbH placed in brine shrimp in an artificial saltwater medium. Short–term toxicity test on embryo and sac-fry stages of zebrafish model: (**f**) Observed development of zebrafish when exposed to the GbH (GbH direct contact and 1:1 medium, i.e., the GbH leeched buffer and E3 medium) for 18–96 hpf; (**f.1**) Observed healthy zebrafish eggs at 48 hpf in contact with the GbH. Photomicrographs showing the histopathology of the skin in rats. Normal appearances of the epithelium in the male (**g**) and female (**g.1**) rats of the control group. Minimal acanthosis of the male (**g.3**) and female (**g.4**) rats. Normal dermis appearance in the control rat (**g.2**) and minimal inflammatory cell infiltration in the treated (**g.5**) group.

The toxicity of the GbH was evaluated at three levels by MTT assay. The first-level assay using L929 cell lines enabled the selection of the appropriate stabilizer, making the GbH non-toxic. The second-level assays determine the cell viability during indirect) contact of the GbH (GbH leachate) with 3T6 cell lines. Finally, in the third level, the inertness of the GbH was proved for direct and indirect (GbH leachate) contact of the GbH using HaCat cells. Various stabilizers, such as quercetin (QU), eugenol (EU), vitamin C (Vc) and sodium metaperiodate (SmP), were subjected to MTT assays at different concentrations (1:0.5, 1:1, 1:1.5 and 1:2) of polydopamine-to-stabilizer ratio (Fig. 4a). Interestingly, reasonable cell viability of ~ 50% at 1:1 ratio of polydopamine to sodium metaperiodate was observed. Thus, the SmP was chosen as a stabilizer for the second-level assays using Polish standards^43^. The MTT evaluation of the toxicity of the biomaterial leachate medium in 3T6 cells revealed its non-toxic properties. Among the ratios of polydopamine to the stabilizer, SmP (1:1,1:2, 1:3, and 1:4) compared here, 1:4 displayed a > 80% cell viability (Fig. 4b), which was further tested in various amounts replacing 100% of the growth medium with the biomaterial leachate medium (Fig. 4c). Even though various percentages (25, 50 and 100%) of the biomaterial leachate medium were replaced, there was no major negative impact of the GbH on the viability of 3T6 cells and it was comparable to the controls, namely PVA-starch hydrogel and the GbH base. The final MTT assay was carried out on the HaCat cell line (Fig. 4d) with two different methods, direct and indirect contact of the GbH (Fig. 4d.1). The cell viability remained unaffected, i.e., 100% in both test methods and the values observed were comparable to the growth controls (Fig. 4d).

#### In vitro model-based toxicity analysis

The effect of the direct and indirect contact of the GbH was explored through in vitro model-based assays using brine shrimp (*Artemia salina*) and zebrafish (*Danio rerio*) embryos. The brine shrimp lethality assay is a rapid test to evaluate cytotoxicity^44^. The GbH immersed in phosphate buffer, incubated at 35–37 °C for 24 h, was used as a test solution for brine shrimp assay. The 24 h hatched nauplii (n = 10) were inoculated in different ratios (1:4, 1:1 and 1:0) of the test solution and artificial seawater. The development of the active young to the naupliar stage with healthy growth in the body size and antenna hair (Fig. 4e–e.5) was indicative of the non-toxic nature of the GbH test solution. The samples of direct contact of the GbH immersed in seawater also showed a similar growth pattern of the nauplius. A 10% mortality rate was observed in 1:0 ratio used in the indirect assay, which ascribes to the presence of low saltwater content. Whereas 15% mortality rate was observed in the direct contact of the GbH immersed samples due to the nauplii tangled to the GbH. (Fig. 4e.5)^45^. The controls maintained in the incubated plates and aerated fish tank displayed similar naupliar growth patterns.

The fish embryo test (FET) assay using zebrafish embryos (n = 10) (Fig. 4f,f.1) was performed similarly to the brine shrimp lethality assay with slight modification using E3 medium^46^. The embryos were observed each day to record any development changes due to their exposure to the GbH directly and the GbH leeched test solution according to the OECD guidelines for testing chemicals No. 212: Fish, short-term toxicity test on embryo and sac-fry stages^47,48^. The apical observations were performed on each tested embryo (Fig. 4f, f.1) every 24 h until 96 h post-fertilization (hpf) and the severity scaling (Table 6) of the toxicity of the GbH on the development of the embryos was recorded. The other observations included eye development, movement, blood circulation and pigmentation, head-body development, pictorial fin and protruding mouth^49^. In addition, the beginning of hatching at ~ 72 h in both the treatment and control groups was documented (Fig. 4f,f.1). The negative results (Table 6) in the severity scaling indicated the healthy development of the zebrafish embryo upon direct and indirect contact with the GbH.

**Table 6:** Apical observations of acute toxicity in zebrafish embryos for 96 hpf.

Observations (n = 10)	Exposure time (h)			
	24	48	72	96
Coagulated embryos	−	−	−	−
Lack of somite formation	−	−	−	−
Non-detachment of the tail	−	−	−	−
Lack of heartbeat	−	−	−	−

−  = No death; +  = Mild; + +  = Moderate; + + +  = High and + + + +  = Severe.

#### In vivo acute dermal toxicity analysis

The acute dermal toxicity experiment was undertaken according to regulatory guidelines of OECD-402 for testing of chemicals^50^. The toxicity range-finding and confirmatory study were performed using selected healthy Wistar albino rats (n = 10) after acclimatization. The test material, GbH, neither produced mortality nor showed any clinical signs of toxicity (Table 7) in the tested in Wistar albino rats throughout the observation period (14 days). Under the test conditions of OECD-402, the experiment did not yield the dermal LD50 of the GbH in rats and the formulation was non-toxic up to 2000 mg/kg body weight (> 2000). The GbH-exposed rats survived the experimental protocol and were sacrificed, as the necropsy was not required (Table 8). Histopathological results (Fig. 4g–g.5) clearly show no sign of inflammation or abnormalities in the applied region of the GbH in the male and female groups.

**Table 7:** Recorded clinical signs and mortality for range-finding study and confirmatory test due to applied GbH.

Parameters	Incidence of clinical signs observed after dosing on																					Mortality	
	Day 0							Day (1-14)															
	Min	Hour																					
	30	1	2	3	4	5	6	1	2	3	4	5	6	7	8	9	10	11	12	13	14	Total	%
Mortality (Total)	0	0	0	0	0	0	0	0	0	0	0	0	0	0	0	0	0	0	0	0	0	0/10	0
Local clinical signs	0	0	0	0	0	0	0	0	0	0	0	0	0	0	0	0	0	0	0	0	0	0/10	
Redness	–	–	–	–	–	–	–	0	0	0	0	0	0	0	0	0	0	0	0	0	0		
Pain	–	–	–	–	–	–	–	0	0	0	0	0	0	0	0	0	0	0	0	0	0		
Swelling	–	–	–	–	–	–	–	0	0	0	0	0	0	0	0	0	0	0	0	0	0		
Systemic signs	0	0	0	0	0	0	0	0	0	0	0	0	0	0	0	0	0	0	0	0	0		

− = Observed after 24 h; 0 = No clinical signs; +  = Mild; + +  = Moderate; + + +  = High and + + + +  = Severe.

**Table 8:** Individual animal fate and necropsy findings.

Animal ID				Fate	Time	Gross findings
Group 1		Group 2				
Male	Female	Male	Female			
20,156-1	20,156-6	20,156-11	20,156-16	TS	Day 15	NAD
20,156-2	20,156-7	20,156-12	20,156-17	TS	Day 15	NAD
20,156-3	20,156-8	20,156-13	20,156-18	TS	Day 15	NAD
20,156-4	20,156-9	20,156-14	20,156-19	TS	Day 15	NAD
20,156-5	20,156-10	20,156-15	20,156-20	TS	Day 15	NAD

*TS:* terminal sacrifice, *NAD:* no abnormality detected, *FD:* found dead.

### In vitro wound healing assay: GbH and scratch closure

**Figure 5:**
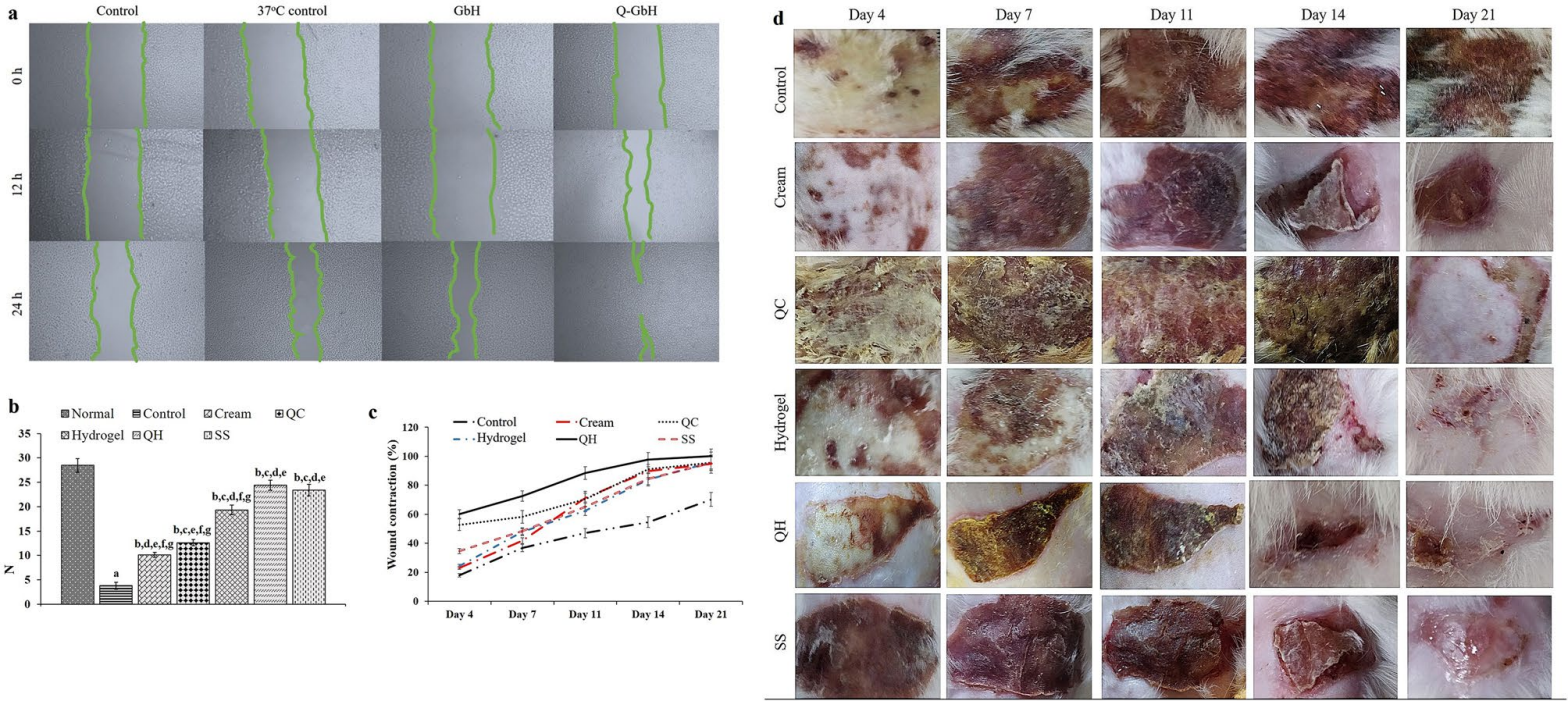
Wound healing performance of GbH: (**a**) Scratch wound healing performance of HaCat cells in 100% replaced of the GbH incubated medium. (**b**) Changes in skin tensile strength in the Normal, Control, cream base group (Cream), quercetin loaded cream (QC), GbH base (Hydrogel), quercetin loaded GbH (QH) and silver sulfadiazine (SS) treated groups. The results are represented as the mean ± S.D. with n = 6 in each group ^a^*p* < 0.05 as compared to the Normal group; ^b^*p* < 0.05, as compared to Control group; ^c^*p* < 0.05, as compared to Cream group, ^d^*p* < 0.05, as compared to QC group, ^e^*p* < 0.05, as compared to Hydrogel group, ^f^*p* < 0.05, as compared to QH group, ^g^*p* < 0.05, as compared to SS group. (**c**) The effect of treatments on burn wound contraction (%) on day 4, day 7, day 11, day 14 and day 21 was a superior and faster wound contraction compared with the control and placebo groups. (**d**) Changes in wound contraction of the normal, control, Cream, QC, Hydrogel, QH and SS treated groups.

The in vitro scratch wound assay demonstrated an efficient cell migration in the scratches in the HaCat cells, which were exposed to the leachate of the GbH and quercetin-loaded GbH in the medium (Fig. 5a). The cell monolayer scratch was partially closed compared with the controls within 24 h of exposure to the biomaterial leachate medium. The images captured at intervals were qualified to prove the non-toxicity nature of the GbH, which favour an efficient cell migration for the wound closure^51^. The observed closing of the artificial gap, ‘the scratch’, on the confluent cell monolayer of HaCat cells, where the cells on the edge of the newly created gap moved towards the opening to close the ‘scratch’. Thus, new cell–cell contacts were again established in the quercetin-loaded and non-loaded GbH (Figure. a. 24 h-Q-GbH). A similar observation of efficient wound closure of in vitro ischemic scratch assay with low levels of immune cell infiltration and cytokine due to quercetin has been reported^52^. Previous reports on the analysis of quercetin-loaded liposomes developed in different amounts of carbopol and gelatin led to an accelerated injury cure, substantially decreasing the wound closing time^53^. Numerous researchers have developed quercetin as a model drug to promote wound healing^54,55^.

### In vivo wound healing assay: GbH and second-degree burn wound closure

The nutritional intake, water consumption and body mass of the Wistar rats having sustained burn injuries remained unaltered. Promptly after the burn injury, the skin on the dorsal region exhibited swelling. The lesions were evident in the form of red patches as of day 4 and necrotic tissues were found to be coated with a crust by day 7.

#### Effect of GbH on the rate of wound healing

An early epithelialization of wounds (Table 9) was observed in the treated groups administered with 1% quercetin GbH-treated group (QH) and silver sulfadiazine (SS). The control group did not show proper wound healing until day 21, whereas the QH and SS exhibited early epithelialization of wounds from day 14 and 16, respectively. The placebo groups (i.e., cream-base treated group (Cream) and GbH-base treated group (Hydrogel) observed early epithelization from day 16 and 17. The placebo groups signify the positive effect of the drug-loaded GbH and -cream formulation.

**Table 9:** Effect of treatments for initiation of epithelialization.

Sl. no	Parameter studies	Epithelialization period (Day)
1	Control	21
2	Cream	16
3	QC	15
4	Hydrogel	17
5	QH	14
6	SS	16

The test tissue specimens were machine clamped and the maximum force generated to tear the specimens indicated the quality of the tissue. A significant difference in tissue tensile strength (Fig. 5b) between control and all treatment groups (*p* < 0.05) was observed. Indicating the need for burn wound care, the tensile strength of the QH and SS groups was 24.4 and 23.37 N, respectively, whereas the control group recorded 3.77 N. A better tensile strength of the QH and SS groups was of a similar range, whereas the placebo group of Cream and Hydrogel base treated showed as 10.1 N and 19.32 N with a significant difference (*p* < 0.05) as compared to the drug-loaded formulation, thus emphasizing the positive role of the GbH in burn wound healing. The recovery of tensile strength of the QH and SS proved the normalization of skin with a tensile strength of 28.45 N by day 21.

The percentage of wound contraction (Fig. 5c,d) were significantly higher (*p* < 0.05) for the QH and SS groups compared to the control group. The outcome of the GbH-mediated wound closure in the second-degree burn on the rat model has proven to be beneficial. The QH-treated animals displayed more substantial wound closing (45.00 ± 1.46% on day 4; 98.24 ± 3.1% on day 14) than the SS treated ones (47.67 ± 1.8% on day 4; 96.4 ± 2.2% on day 14) and the untreated control group (18.00 ± 2.5% on day 4; 54.52 ± 1.25% on day 14) on day 4 and day 14, respectively (Fig. 5,d). The gross morphological studies showed decreased inflammation and redness and limited scarring in the QH group on day 14. Also, the epidermis rejuvenated to the normal skin architecture in the QH and SS treated wounds, while signs of inflammation and premature wound closure were observed in the control groups on day 21 (Fig. 5d)

#### Biochemical profile of healed wounds

**Figure 6:**
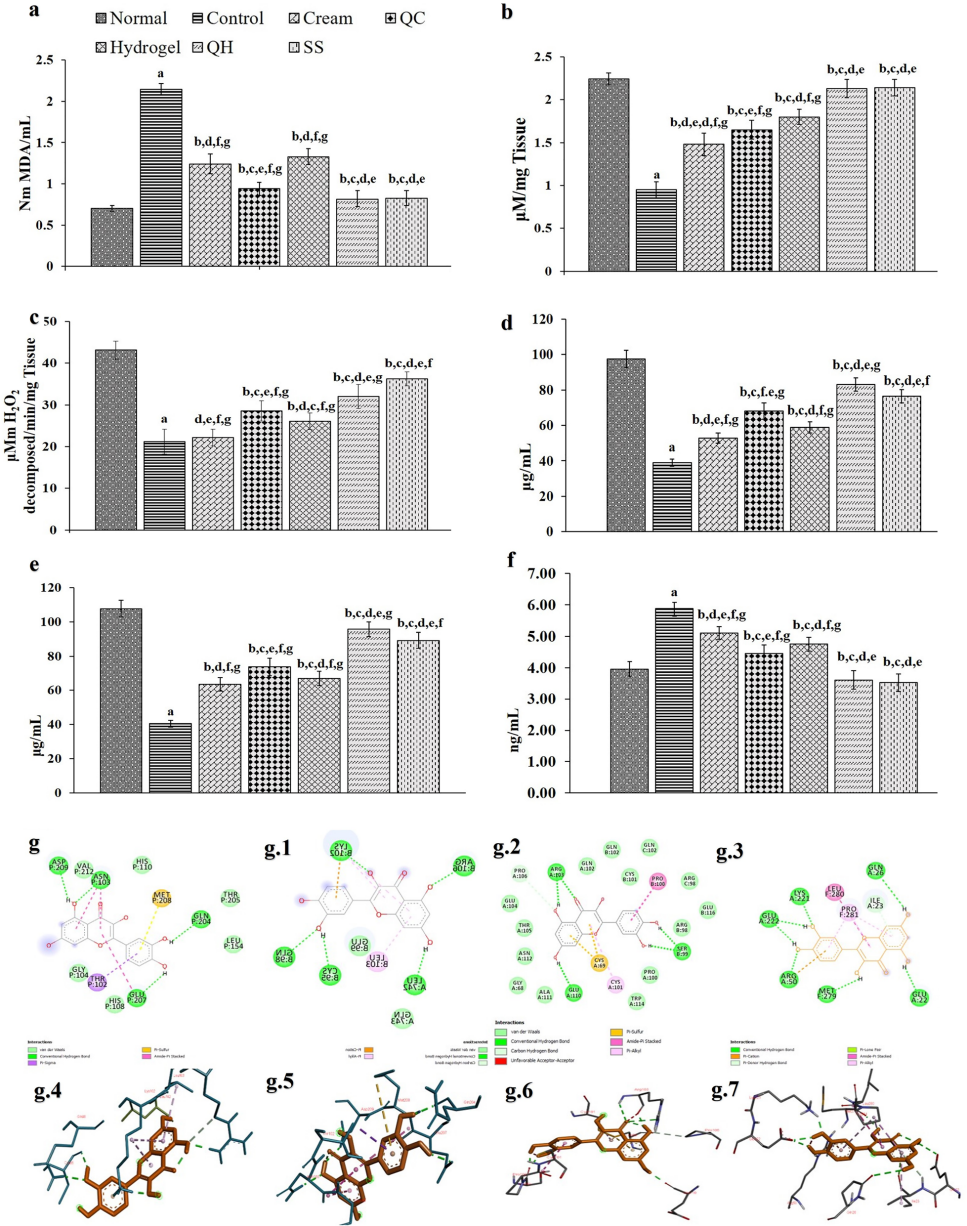
In vivo wound healing changes in (**a**) MDA; (**b**) GSH; (**c**) CAT; (**d**) HXP; (**e**) HXA and (**f**) NF-κB levels of the Normal, Control, cream base group (Cream), quercetin loaded cream (QC), GbH base (Hydrogel), quercetin loaded GbH (QH) and silver sulfadiazine (SS) treated groups. The results are represented as the mean ± S.D. with n = 6 in each group ^a^*p* < 0.05 as compared to the Normal group; ^b^*p* < 0.05, as compared to Control group; ^c^*p* < 0.05, as compared to Cream group, ^d^*p* < 0.05, as compared to QC group, ^e^*p* < 0.05, as compared to Hydrogel group, ^f^*p* < 0.05, as compared to QH group, ^g^*p* < 0.05, as compared to SS group. 2-D and 3-D image of quercetin interaction with: (**g**) and (**g.4**) 1SVC target interaction; (**g.1**) and (**g.5**) 3BRV target interaction; (**g.2**) and (**g.6**) 1NFI target interaction, and (**g.3**) and (**g.7**) 2E7A target interaction.

The malondialdehyde (MDA) levels (Fig. 6a) of the QH treated tissue samples were recoded as 0.814 nmoles MDA/mL and were lower than the control (*p* < 0.05) group with 2.147 nmoles MDA/mL, whereas normal skin was recorded as 0.705 nmoles MDA/mL. A substantial increase (*p* < 0.05) in the control group MDA is a sign of lipid peroxide damage, which signifies to free radical damage, leading to incomplete wound healing by day 21. The MDA levels of QH and SS treated groups were 0.814 and 0.827 nmoles MDA/mL, respectively and identical to each other and comparable to normal skin tissue. The lowered MD levels in the QH treated samples indicate the quercetin’s effect in reducing the lipid peroxidation and antioxidant protection during burn wound healing. Antioxidant protection against burn-induced oxidated injury by using oral and topical treatment of Myrtle (*Myrtus communis*) has been previously reported^56^. Thus, the reversal of biochemical indices is attributed to anti-inflammatory compounds’ potential antioxidant effect.

Both non-enzymatic and enzymatic antioxidants, i.e. glutathione (GSH) and catalase (CAT), respectively, play a prominent role in dynamically regulating the levels of reactive oxygen and its intended harm, favouring wound healing^57,58^. In the control samples, the GSH (Fig. 6b) and CAT (Fig. 6c) levels were recorded as 2.24 μ mol/g and 14.83 µ moles of hydrogen peroxide utilized/mg/tissue/min, respectively. A significant reduction in GSH (Fig. 6b) and CAT (Fig. 6c) levels were observed in the control group when compared to the treated and normal groups (*p* < 0.05). The QH and SS treated groups aimed at restoring regular tissue function of GSH (2.13 and 2.14 μ mol/g tissue, respectively) and CAT with (22.41 and 25.341 µ moles of hydrogen peroxide utilized/mg/tissue/min) that is comparable to that of the normal group with 2.24 μ mol/g tissue GSH and 30.182 µ moles of hydrogen peroxide utilized/mg/tissue/min of CAT (Fig. 6b and c). The QH significantly restored (*p* < 0.05) GSH and CAT levels by day 21, when compared with the placebos (GSH: 1.48 and 1.8 μ mol/g, and CAT: 15.533 and 18.207 µ moles of hydrogen peroxide utilized/mg/tissue/min for Cream and Hydrogel groups) and 1% quercetin cream-base treated group (QC) with 1.65 μ mol/g tissue and 28.554 µ moles of hydrogen peroxide utilized/mg/tissue/min, respectively.

#### Connective tissue profile of healed wounds

The hydroxyproline (HXP) and hexosamine (HXA) levels in test tissues can be correlated to the healing observed. The HXP is the critical element of collagen, whereas the HXA is the ground substrates for constructing connective tissue components^59^. Thus, the HXP and HXA levels represent a perfect candidate for a connective tissue marker in wound healing. During the healing process, the HXP and HXA contents increased in all treated groups relative to the control group (Fig. 6d and e). The HXP and HXA levels of the burn group without any treatment were 38.911 and 40.477 μg/mL, respectively, which was significantly lower (*p* < 0.05) compared to that of the normal group with 97.546 and 107.752 μg/mL, respectively. The QH and SS groups restored their HXP and HXA functional levels to 83.053 and 76.343, and 95.698 μg/mL and 89.22 μg/mL, as observed in the normal group with 97.546 and 107.752 μg/mL by day 21. The QH substantially restored (*p* < 0.05) the levels of the HXP and HXA compared with the placebo groups (HXP: 52.858 and 58.729 μg/mL, and HXP: 63.559 and 66.898 μg/mL for Cream and Hydrogel groups) and QC (HXP: 68.160 and HXA: 73.785 μg/mL) groups as on day 21. Interestingly, the SS group recoded 76.343 and 89.221 μg/mL of tissue HXP and HXA levels, similar to the QH group. The substantial decrease in the HXP and HXA levels in the control tissue content indicates low collagen levels, insufficient connective tissue formation, and an inadequate wound healing process. Thus, the observation in the QH treated group highlights the intended healing property of the formulation.

#### Restored NF-κB levels of healed wounds

The functions of the NF-κB are several in wound healing as it modulates inflammation and cell survival and promotes the remodelling of cell junctions and the assembly of a cytoskeletal structure around the wound^60^. The NF-κB levels of the control group were 5.88 ng/mL and were significantly high (*p* < 0.05) relative to the normal group with 3.95 ng/mL, implying inflammation persistence. The QH and SS treated groups restored their usual NF-κB functional levels and were observed at 3.61 and 3.52 ng/mL, similar to the normal group (Fig. 6f) tissue NF-κB levels. The QH substantially restored (*p* < 0.05) NF-κB levels compared with the placebos (5.11 and 4.75 ng/mL of tissue NF-κB levels of Cream and Hydrogel groups) and QC treated groups (4.46 ng/mL tissue NF-κB levels) as on day 21. Thus, the results emphasize the effect of the GbH in facilitating wound healing and restoring the expected functioning of the NF-κB akin to a normal skin tissue.

##### In silico exploration: quercetin and anti-inflammatory targets

The transcription factor NF-κB and the multifunctional cytokine TNF-α are potent inflammatory mediators that actively play predominant roles in cellular events such as cell survival, proliferation, differentiation, and death^61^. Researchers have well-established quercetin as a model medication for improving wound healing^53,56^, and the current study highlights the beneficial effects of the quercetin-supplemented GbH in the recovery of second-degree burn wounds in rat models. The in silico analysis of quercetin against the potential inflammatory mediator targets (Fig. 6g–g.8 and Table 10) observed an excellent binding score and hydrogen bonding to multiple amino acids. The compound binds at the p50 domain of the protein 1SVC, which translocates to the nucleus, where it binds to the DNA (Fig. 6g,g.4). The coiled-coil region of 3BRV lies between positions 49–356 (Fig. 6g.1,g.5). Many coiled coil-type regions are involved in critical biological functions, such as the regulation of gene expression. The region between 44 and 111 of 3BRV actively participates in the interaction with CHUK/IKBKB, thus enabling IKK-mediated phosphorylation of RelA/p65, which overall actively promotes an inflammation response through NF-κB activation. The domain region of 1NFI (Fig. 6g.2,g.6) between 19 and 306 functionally regulates the RelA target gene promoter accessibility. Quercetin forms hydrogen bonds with amino acids in the 1NFI domain region, rendering it functionally inactive. Mutagenesis at positions 105 and 108 of protein 2E7A (Fig. 6g.3,g.7) has led to low activity and inactivity^62^. The current docking highlights the binding of quercetin in the active region of 2E7A.

**Table 10:** Binding score (kcal/mol) of quercetin and targets of inflammatory mediators.

Targets	Protein name	Binding score (kal/mol)	H-bonds
1SVC	NF-κB p50	− 6.57	ASP208, ASN103, GLN204, GLU207
3BRV	IKK complex	− 5.19	LYS102, ARG106, CYS95, LEU742
1NFI	IκBα	− 6.5	GLU222, LYS221, GLN26, ARG50, MET279, GLU22
2E7A	TNF receptor subtype	− 6.14	ARG103, SER99, GLU110

#### Histological assessment

**Figure 7:**
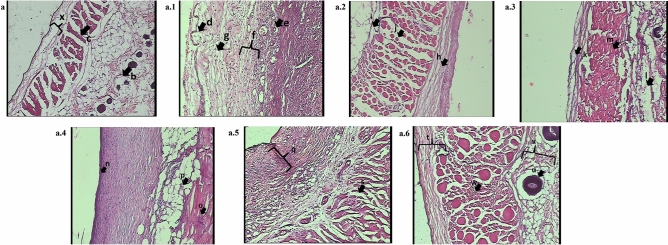
Burn wound histopathological assessment on day 21: Standard morphology of the epidermis, dermis and hypodermis was found during photomicrographs of a histopathological examination of the Normal group (**a**). The Control group (**a.1**) was observed to have marked characteristics of burn crust development in the dermis and epidermis, extreme leukocyte aggregation, congestion of the blood vessels, hair follicles, and the sebaceous glands. Moderate leukocytes, blood vessel destruction, and degenerating degeneration of hair follicles and sebaceous glands were observed in the topical application of the Cream and the GbH (Hydrogel) placebo group (**a.2**) and (**a.3**). The quercetin drug-treated cream and the GbH group (QC, QH) and silver sulfadiazine (SS) (**a.4**), (**a.5**) and (**a.6**) displayed no symptoms of leukocyte accumulation, favoured by epithelium regeneration. The restored skin architecture refelects the regeneration of the epidermis upon the topical application of quercetin loaded GbH, which is in accordance with normal skin.

The normal skin (Fig. 7a) shows an architecture with a well-defined epidermis and dermis layer in the histopathological evaluation. The epithelial layer (x) is intact with no signs of inflammation. The dermis has many sebaceous glands (b) and well-defined collagenous fibres (c) in the upper layer of the dermis. In the control group (Fig. 7a.1), there is a complete loss of the epidermis and substantial destruction of the superficial skin layers (d). Vacuolar cytoplasmic disintegration in the basal cell layer is prominent (e) along with neutrophilic infiltration to the injury site (f). The skin shows coagulation of the epidermis and dermis, and the denatured collagen appears swollen (g), homogeneous and infiltrated by exudative cells. The placebo group, Cream base treated group (Fig. 7a.2), displayed a burn healed tissue with moderate inflammation, with no signs of epithelial or dermal regeneration (h). The basal cell layer remains degenerated (i) and markedly vacuolated, indicating impeded healing. The collagen fibres remain degenerated (j) with no signs of microvascular regeneration. Similarly, the placebo group, Hydrogel base treated group (i.e., the GbH) (Fig. 7a.4), exhibits epithelium with some signs of regenerative growth (k). A marginal decrease in the vacuolization of the basal cell layer (l) indicates healing of the tissue, and a decrease in the swelling of collagen fibres is evident (m).

The drug-loaded cream-treated group, i.e., QC (Fig. 7a.3), aimed at regenerating the epidermis and dermis layers, where their differentiation is evident (q). Significant architectural restoration of the basal cell layer is seen in the form of markedly reduced oedema and negligible signs of vacuolization (r). However, healing of the dermis layer remains incomplete with the presence of small vacuoles in collagen fibres (s). A significant epithelium regeneration can be observed in the drug-assisted GbH treatment group, i.e., QH (Fig. 7a.5). The differentiation in the epidermis and dermis layers appear almost restored to a normal histological architecture (t). The presence of sebaceous glands (u) indicates a physiological restoration of skin tissue function. The basal cell layer appears normal in shape and architecture, with no signs of inflammation (v). The collagenous fibres in the dermis show no signs of swelling or other inflammatory characteristics (w). The positive control of the SS cream-treated group (Fig. 7a.6) shows evident epithelium regeneration (n). The basal cell layer displays restorative changes, evidenced by a significantly decreased vacuolization (o). However, swelling and oedema in collagen fibres (p) can still be observed at low levels.

## Discussion

The skin is the largest and essential organ of our body, which is an outer defensive layer. Damage to the skin affects the various functions and compromises the patient’s working capacity and independence. A burn injury is a traumatic experience and depending on the severity of the wound, slow wound healing, infection, pain, and hypertrophic scarring remain a major challenge in burn research and management^63^. A burn wound experiences high capillary permeability due to direct exposure to heat, which causes the plasma to leak out to the interstitial place from the capillaries. During a burn condition, an increased plasma leak and capillary permeability persist till 48 h and is maximum in the initial 8 h. Burn wounds are sensitive and the patient experiences pain depending upon the severity of the wound. The advent of plasma leak and generalized capillary permeability is a phenomenon specific to burn wounds^64^. The current research focuses on soft wound dressing that is sticky due to the bioinspired property of polydopamine. Such soft wound dressing can be beneficial for treating burn wounds since the stickiness of the hydrogel is not extreme adhesive and tough. For many marketed gelatin-based sealants, tissue adhesives can be beneficial for tissue repairs^1,8,18^. Such tissue injuries call for immediate attention to restore standard organ functionality. In major injuries associated with trauma or surgeries, tissue adhesives are expected to be strong, robust, elastic, non-toxic and perform diverse surface adhesion^8^.

**Figure 8:**
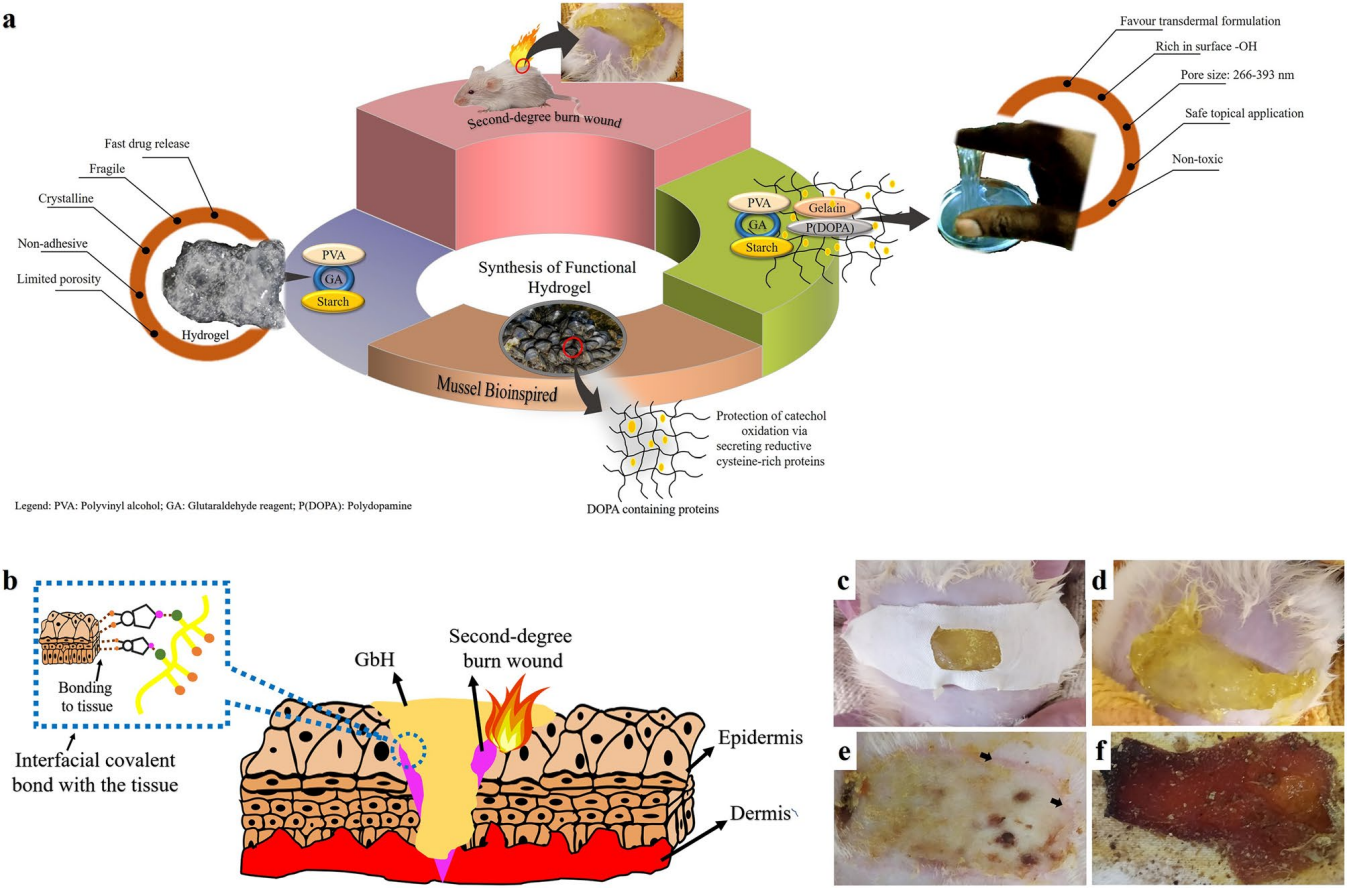
Highlights of the GbH in the current research: (**a**) Development of bioinspired gelatin-based diverse surface sticky hydrogel for second-degree burn wound care; (**b**) Schematic of the surface interaction of the catechol group with the burn wound area; (**c**) soft wound patch interaction with wound tissue; soft wound patch; (**d**) QH placed on second-degree burn wound; (**e**) Observable wound repair on day 3 (arrow) of dressing and (**f**) Absorption of exudates: a hallmark of the ideal hydrogel.

The hydrogel blend prepared by the solution casting method was translucent and exhibited no sticky property (Fig. 8a). The blend of PVA-starch and crosslinked with GA served as a base to further incorporate the sticky property. The recent bioinspired research tends to modify the polymeric properties of hydrogels^2,18^. The practice of using alkali polymerized dopamine^65^ to induce diverse surface sticky properties in a polyacrylamide and bis-acrylamide hydrogel system has been previously reported^4^, even though various researchers have established its carcinogenic effect^66^. The present research aims at a highly biocompatible composition and a cost-effective bioinspired hydrogel that can be sticky onto diverse surfaces using alkali polymerized dopamine. The physical property estimates of the GbH showed the good swelling property in different solvents that mimicked the physiological condition of the wound (Fig. 8a). Polyelectrolyte-based hydrogels, due to charge repulsion on the polymer chains, prove beneficial for achieving controlled drug release^29,30^. Burn wounds exhibit more significant fluid loss of 5000 g/m^−2^/d^−1^ compared to normal wounds. The current composition of the GbH reports low WVT and high MRC values and is comparable to the control^31^. The essential parameters of swelling percentage WVT, MRC and GF enable exudate absorption and reduce the water transfer from the wound. Thus, ensuring a condition for effective wound healing by providing proper drug diffusion.

In vivo dermal wound dressing, the GbH (Table 11) demonstrated accelerated skin tissue regeneration. The GbH could be easily removed without leaving any residue or pain on the rat model. The sticky GbH confirms its diverse surface performance. The free catechol group of polydopamine interacts with amine groups of the tissue surface (Fig. 8b)^25^. Burn wound healing remained constant in the control group from day 1 to day 7. On the other hand, the scale of healing was prevalent in the majority of burn wound treated groups as of day 3. The histopathological analysis of almost every treatment group showed healing of burn wounds, but the QH demonstrated a better and faster healing process than the cream formulation treatment group and was similar to the SS treated group. The GbH formulations have an advantage over the cream formulations since the former can be replaced by new formulations every third day, whereas the cream formulation must be applied daily. The GbH as a soft wound dressing (Fig. 8c) was not painful to the rats and there was no significant redness or change in the dietary routine of the rats owing to the infliction of a second-degree burn wound or GbH dressing (Fig. 8d).

**Table 11:** The optimized composition of GbH.

Sl. no	Components	Concentration
1	PVA	10%
2	Starch	5%
3	GA reagent (Ethanol: GA: HCl)	10.55 mL for 100 mL
4	Polydopamine	0.5 μL
5	Gelatin	20% (500 μL gelatin for 10 mL of hydrogel)
6	Sodium metaperiodate	1:5 (Polydopamine: Sodium metaperiodate)

Patent application no: 202041044794, Government of India patent office, Status: Filed.

A remarkable finding of the present investigation is the development of pink and fresh cells (Fig. 8e) along the wound boundary on day 3 with the quercetin supplemented GbH treatment (QH group), which was not the case with any other treated group. A recent finding of the quercetin dosage in promoting wound healing favoured an inhibition of mitogen-activated protein kinase and NF-κB activation in the in vitro cell scratch assay, which was conducive to the healing of pressure lesions^52^. Similarly, the placebo group also confirmed the significant effect of the quercetin-loaded GbH on healing the second-degree burn wound in the present research work. Previous reports on an analysis of quercetin-loaded liposomes developed in different amounts of carbopol and gelatin led to an accelerated injury cure, substantially decreasing the wound closing time^53^. Numerous researchers have developed quercetin as a model drug to promote wound healing^54,55^. Likewise, the current research also emphasizes the positive impact of quercetin-supplemented GbH in healing second-degree burn wounds in rat models. In order to promote the healing of wounds, the properties of hydrogel to absorb exudates and maintain a clean, moist, healing environment (Fig. 8f) were some of the other highlights of the GbH in the present investigation.

It is essential to consider that wound healing is a complex process that involves various factors, such as a moist and warm environment. Thus, as centred on the ‘wet wound healing theory’, a moist healing condition is ideal for the development of granulation tissue and fostering the separation of dermal cells. The GbH formulation recognizes such crucial elements and facilitates rapid wound healing by absorbing excess exudates and allowing for the exchange of oxygen and water vapour. It also guards against microbial invasions. The GbH, formulated in the present investigation, is non-toxic, non-allergenic, convenient and cost-effective. It has a controlled drug delivery feature and properties enabling it to stick onto different surfaces, like medical implants and wet or dry wound surfaces. The GbH may be used as a preventive anti-infection patch to control bacterial or fungal infections on the skin. The developed GbH was validated as an ideal wound dressing material for second-degree partial burn wounds in rat models. The above findings culminate in the fact that the bioinspired formulation of the GbH can improve wound healing and skin repair of second-degree burn wounds in rat models, thus validating its pre-clinical use.

## Method

A detailed description of the materials and methods is available in SI Materials and Methods.

### In vivo acute dermal toxicity studies

#### Animals and experimental plan

The experiment was carried out at the Institute for Industrial Research & Toxicology, F-209, U.P.S.I.D.C., M.G. road, Ghaziabad-201302, India and the experiment was labelled as Project No.: 202112-25; Report No: IIRT/TOX/202112/ADT/0112; Date: 14–12-2021. All methods were carried out in accordance with guidelines and regulations of Committee for the Purpose of Control and Supervision of Experiments on Animals (CPCSEA), New Delhi, India. The methods implemented in the current study are in accordance with ARRIVE Guidelines 2.0^67^. A protocol detailing the acute dermal toxicity studies, treatment groups and design of the experiment is mentioned in SI Materials and Methods. The protocol for dermal toxicity study was for 14 days and animals were euthanized by isoflurane overdose using small animal anesthesia system.

### In vivo second-degree burn wound healing studies

#### Animals and experimental plan

Rats (either sex), weighing between 250 and 300 g, were procured from the Disease-Free Small Animal House Facility (DFSAH) of Lala Lajpat Rai University of Veterinary and Animal Sciences (LUVAS), Hisar, Haryana, India. All methods were carried out in accordance with guidelines and regulations of Committee for the Purpose of Control and Supervision of Experiments on Animals (CPCSEA), New Delhi, India. The methods implemented in the current study are in accordance with ARRIVE Guidelines 2.0^67^. The animals were quarantined and housed at the Central Animal House Facility (CPCSEA Registration no. 1753 Wistar /PO/E/S/14/CPCSEA) for acclimatization for seven days before experimentation. The experimental animals were divided into seven groups (n = 6), and the protocol duration was 21 days. After 21 days the animals were euthanized by isoflurane overdose using small animal anesthesia system. A protocol detailing the induction of second-degree burn injuries, treatment groups and design of the experiment is mentioned in SI Materials and Methods.

### Pathological examination

#### Pharmacological parameters

The animals were anaesthetized and sacrificed by decapitation, and skin tissue samples were collected and processed according to the standard protocols. The samples used for histological observation were deposited in 10% neutral buffered formalin. Constructive pharmacological criteria, including time of epithelialization and wound contraction, have been monitored by regular protocols^68^. The quality of the burn-healed skin, when compared with a normal group, was evaluated using a wound stretching machine, EFG500E, EFGE digital force gauge. The histology studies began immediately on day 21, right after the induction of the burn injury.

#### Biochemical evaluation of burn wound

After decapitation on day 21, the full thickness of the healed and natural skin (1 cm^2^) was cautiously removed. The tissue was weighed and then homogenized with a glass homogenizer at 4 °C in 1 × PBS (tissue weight (g): PBS (mL) volume = 1:9) and followed by centrifugation at 10,000 g at 4 °C for 30 min. The biochemical parameters include malondialdehyde (MDA)^69^, glutathione (GSH)^70^ and (catalase) CAT^71^ levels of the homogenized skin sample, according to standard protocols, which were analyzed using UV/Visible double beam spectrophotometer (Shimadzu), and expressed in nmoles MDA/ml, μ-mol/g tissue and µmoles of hydrogen peroxide utilized/mg/tissue/min, respectively.

#### Connective tissue evaluation of burn wound

The hydrolysate of the skin tissue was prepared according to the previously mentioned protocol of day 21^72^. The hydroxyproline (HXP) and hexosamine (HXA) contents of granulated tissues were estimated using UV/Visible double beam spectrophotometer, Shimadzu, according to previously mentioned protocols and expressed in μg/mg of tissue.

#### Estimation of NF-κB in healed burn wounds

The nuclear factor kappa B (NF-κB) of the homogenized skin sample was measured on the basis of the Sandwich-ELISA theory using Rat NF-ŚB ELISA Package (Biolab Technology Laboratory, Shanghai Korian Biotech Co., Ltd). The sample was prepared following the operator’s protocol and measured at 450 nm within 10 min using a microplate reader (Alere AM 2100).

### Histopathological evaluation and Statistical analysis

A rat from each group was euthanized on day 21 after the wounding for histopathological examination. Samples of tissue (2 × 3 mm) placed in buffered formalin (10%), dehydrated by alcohol, have been excised and eventually inserted in paraffin wax blocks. For the assessment of pathological modifications, thin pieces of tissue samples (5 μm) were stained with hematoxylin and eosin (H and E)^73^. The stained slides were studied under an Olympus CX41 microscope, Olympus Life Science Solutions, using Magnus Pro Image Analysis software. Slides were investigated to determine congestion, degeneration and necrosis, neovascularization, fibroblast proliferation and epithelialization, oedema and leukocyte infiltration. All the statistical results were analyzed using a two-way ANOVA method followed by Tukey’s post-hoc analysis with *p* ≤ 0.05 considered significant for all values on GraphPad Prism 8.4.3.686.

### Ethical approval

1. The research protocol for acute dermal toxicity of bio-inspired gelatin-based adhesive hydrogel in Wistar albino rats as per regulatory guidelines of “OECD 402” for testing of chemicals was carried out at Institute for Industrial Research & Toxicology, Ghaziabad, India, Project No.: 202112-25; Report No.: IIRT/TOX/202112/ADT/0112.
2. The research protocol for second-degree burn wound healing was approved by the Institutional Animal Ethics Committee (IAEC) of Khalsa College of Pharmacy, Amritsar, Punjab. vide approval no. IAEC/KCP/2020/008.

### Patent

Suchithra T. V., Benu G, Gelatin-based adhesive hydrogel as wound dressing patch for diverse surfaces. (Application No: 202041044794, Government of India patent office, Status: Filed).

## Supplementary Information

Supplementary Information.

## Data Availability

All data generated or analysed during this study are included in this published article.
