# Supplementary material for: Bioinspired gelatin based sticky hydrogel for diverse surfaces in burn wound care
Source: Sci Rep. 2022 Aug 12;12:13735. doi: 10.1038/s41598-022-17054-w (PMC9374690; doi:10.1038/s41598-022-17054-w)
Supplement: Supplementary file 1 — Supplementary Information. [file 41598_2022_17054_MOESM1_ESM.docx]

**Bioinspired gelatin based sticky hydrogel for diverse surfaces in burn wound care**

Benu George^1^, Nitish Bhatia^2, 3^, Abhitinder Kumar^2^, A. Gnanamani^4^, R. Thilagam^4^, S.K. Shanuja^4^, Kannan Vadakkadath Meethal^5^, Shiji T M^5^, Suchithra TV^1*^

^1^ School of Biotechnology, National Institute of Technology Calicut, Kozhikode, India

^2^ Department of Pharmacology, Khalsa College of Pharmacy, Amritsar, Punjab, India

^3^ Scool of Medicine and Allied Sciences, G. D. Goenka University, Haryana, India.

^4^ CSIR-Central Leather Research Institute, Adyar, Chennai, India

^5^ Division of Biochemistry and Molecular Biology, Department of Zoology, University of Calicut, Kozhikode, India

*Corresponding author: drsuchithratv@nitc.ac.in

**Supplementary Material**

**Materials and methods**

All chemicals, including culture media, components, and other reagents, were purchased from Sigma-Aldrich (India), HiMedia Laboratories (India), etc. The brine shrimp (*Artemia salina*) and the zebrafish (*Danio rerio*) were obtained from local breeders of Calicut, Kerala, India.

**Synthesis of hydrogel**

The hydrogel blend was prepared by mixing solutions of 10% (w/v) polyvinyl alcohol (PVA) and 5% (w/v) starch in a 1:1 ratio with water and heated with continuous stirring to form a homogeneous mixture. The crosslinker glutaraldehyde reagent (GA) was prepared by combining 0.5 mL of glutaraldehyde in a solution mixture of 10 mL of ethanol and 0.05 mL of hydrochloric acid (HCl). The mixture was then subjected to sonication (bath sonicator) for 30 min and poured into a casting mould, as per the conventional solution casting process for polymerisation, for 12 h at 22 ± 3°C room temperature (RT) and a humidity level of 50-57%. Precautions were taken to avoid the trapping of air bubbles while mixing ^1^.

**Incorporation of stickiness and stabilising property**

In 90 mL of deionised water, 180 mg of dopamine hydrochloride (Sigma-Aldrich) was dissolved under vigorous stirring. Drop wisely, 760 μL of 1 N sodium hydroxide (NaOH) solution was added to the dopamine hydrochloride solution at 50°C until the colour changed to dark brown was indicative of polydopamine ^2^.

A classical optimisation was done to synthesise a sticky hydrogel by adding polydopamine ^2^ and gelatin into the PVA-starch mixture. During the GA integration, the hydrogel was exposed to varying amounts of polydopamine, ranging from 0.5 to 3%, and poured into the casting mold. Later, when the hydrogel was semi-polymerised, i.e., after 4 h, gelatin and sodium metaperiodate were added as stabilisers. A volume of 10 mL of hydrogel was treated with two concentrations of 20% (w/v) of gelatin (500 μL and 1000 μL). Polydopamine was applied in varying amounts of sodium metaperiodate, from a ratio of 1:0.5 to 1:5, and was tested for sticky retention.

**Washing of gelatin-based hydrogel (GbH)**

The GbH is subjected to a washing process to remove excess GA reagent ^3^, and the process was optimised with an effective buffer to ensure the removal of excess aldehyde. The buffers used were Tris-HCl (3 M), phosphate-buffered saline (PBS) (0.1 M), aluminium chloride (AlCl_2_) (50 mM) and distilled water for the Benedict test ^4^. After the selection of washing buffer, gel preservation was also studied.

**Physical evaluation of GbH**

Each physical evaluation (i.e., elongation, diverse surface, swelling, water vapour transmission, moisture retention capacity and gel fraction) was conducted with the GbH and non-drug-loaded GbH. The drugs incorporated were ciprofloxacin, 5-flucytosine and quercetin with different concentrations of drug (a minimum of 5 mg/mL and a maximum of 20 mg/mL).

**Elongation Test**

The elongation of the GbH and two types of patches (Patch-A, the GbH was prepared and dispersed in salicylic acid and patch-B, where the GbH was immersed in 10 mL of salicylic acid solution in acetone) was determined in the universal testing machine (UTM), Shimadzu. The following test conditions were maintained: cross-head speed: 35 mm/min, temperature: 25°C and relative humidity: 55%. Slices of constant dimension (*lbw*; 5×1.5×0.4 cm) were used in the elongation testing.

**Diverse surface test**

The bio-inspired GbH promoted sticky properties on varied surfaces and exhibited a strong elongation. The GbH was placed on a different dry surface (glass, plastic and aluminium) and wet surface (pork and chicken) at RT, overnight. The pull length of each GbH was observed from the respective surface.

**Swelling test**

The hydrogel formulation that promotes osmotic flow and exerts repulsion on the polymer network exhibits a high swelling and hydrophilic property. The GbH was cut into 1cm^2^ and weighed (W_D_). The GbH was incubated for 24 h in water, sodium chloride (NaCl), magnesium chloride (MgCl_2_) and blood (Poultry blood) as it partly mimics the physiological conditions of injury. After incubation, the solution was decanted, and the GbH surface was mopped with filter paper to eliminate any droplets and weighed again (W_S_). The swelling activity was calculated by using the formula ^5^

Swelling ratio (%) = (W_S_-W_D_/W_D_) ×100

**Water vapour transmission test**

An ideal wound dressing should reduce the possibility of fluid loss from the wound surface and provide a safe, moist condition for healing. The GbH was used to close a glass bottle with an opening of 30 mm in diameter, using a Teflon tape (to block the air passage) filled with 10 mL of deionised water. The surface area of the bottle mouth (A) was noted and the bottle was weighed (W_I_) and placed in a hot air oven for 24 h at 40°C. After 24 h, the bottle was removed from the hot air oven and measured again (W_T_). The rate of transmission of water vapour was calculated using the formula ^6^

Water vapour transmission (WVT) = (W_I_-W_T_)/(A×24) (g/m/m^2^h)

**Moisture retention capacity test**

A wound can cause water loss and increase body temperature and metabolic rate. The GbH was uniformly cut into pieces and weighed (W_I_), which was later put into a hot air oven at 40°C for a period of 6 h. After incubation, the GbH was weighed (W_T_) and its moisture retaining potential (MRC) was determined using the formula ^7^,

MRC = (W_I_/W_T_) ×100

**Gel fraction test**

A hydrogel with a good gel fraction (GF) exhibits strong polymerisation efficiency and does not melt in a solution. The GbH was sliced as 1 cm^2^ and put into a vacuum oven until the constant weight was achieved. These samples were measured (W_I_) and submerged in distilled water for four days. After four days, the water was decanted and the sample was again put into the vacuum oven to achieve a steady weight (W_T_). The gel fraction was measured using the formula ^8^,

GF (%) = (W_I_/W_T_) ×100

**Characterisation of GbH**

The GbH sample was freeze-dried in preparation for the scanning electron microscope (SEM) analysis. The surface morphology of the GbH and patches was studied using the SEM, Hitachi SU6600, at various magnifications. The surface functional group investigation of hydrogel and the GbH was carried out by using Fourier-transform infrared spectroscopy (FT-IR), Jasco FT/IR-4700. The spectra were recorded by an FT-IR spectrometer, equipped with an attenuated total reflection unit in the range of 450-4000 cm^-1^. The material's crystallinity was identified by X-ray powder diffraction (XRD) plots. The 2θ peaks of the test and control differ from each other and can be compared with studying the change in the material composition. The patches were subjected to XRD, Miniflex 600 and they used CuKα radiation generated at 40 kV and 40 mA. The range of the diffraction angle was 10.00-70.00^O^2θ. The crystallisation behaviour of the polymeric GbH was analysed by differential scanning calorimetry (DSC), TA Instruments Q50 thermograms. The temperature and energy scales were calibrated by standard procedures. The melting studies were performed in the temperature range of 25-350°C, at a heating rate of 10°C/min, in the N_2_ atmosphere.

**Evaluation of drug release**

**Spectrophotometric method**

- Patches: The drug release profile was assessed using salicylic acid as a 1% drug dosage. Two approaches were implemented: patch-A, where the GbH was prepared and dispersed in salicylic acid; patch-B, where the GbH was immersed in 10 mL of salicylic acid solution in acetone until the solvent was evaporated entirely.

Periodically spectrophotometric values of salicylic acid drug release are noted, which are mathematically expressed to designate the drug release pattern ^9^. The drug-loaded patch-A and -B was put into separate 250 mL beakers containing 100 mL of distilled water and was stirred with a magnetic stirrer. Samples were periodically withdrawn from the beaker using a 1 mL pipette, where the withdrawn quantities were replaced with distilled water. The volume removed was then measured spectrophotometrically at 530 nm, using an acidic ferric chloride solution as a colourant.

**Theoretical validation of drug release profile: Mathematical kinetics**

The kinetic models describe the release of drugs from the dosage forms. The mathematical kinetic models provide a good understanding of the qualitative and quantitative changes in the developed formulation, thus reducing extensive bio-studies ^10^. The *in vitro* drug dissolution data is considered for the prediction of *in vivo* performance and rationale of control release formulation. In this regard, the spectrophotometric values of salicylic acid drug release were used to calculate the controlled drug release percentage for a period of 8-12 h and graphs are plotted for zero-, first-order and Higuchian kinetics to understand the nature of formulation type of drug release pattern.

**Agar well diffusion method**

The approach is usually based on the diffusion pattern of the formulation (patch-A and -B), where the drug transfer occurs from a higher concentration to a lower concentration ^11^. A 3% agar solution was made with vigorous stirring and boiled for 15 min. Later allow it to cool to 50°C and pour 30 mL of agar solution into each Petri dish and add 1 mL of 5% ferric chloride (or) ferric nitrate. Then mix and allow solidifying, draw wells of equal dimension and place the salicylic acid loaded-GbH in the well. The violet/purple colour formed on agar plates is determined by a reaction between the salicylic acid and ferric chloride. The evaluation of the drug release pattern from the GbH in the agar well is analysed by plotting the width of the zone formed (violet/purple width) on the agar plates vs. time.

**Assessment of drug diffusion from GbH**

**Agar diffusion method**

The drug-loaded GbH was used to study drug diffusion and bioactivity. The GbH and the drugs (mixed in dimethyl sulfoxide (DMSO)) were in the ratio of 1:2 and 2:1. Later, 1 cm^2^ piece of 0.5 mL drug-loaded GbH (ciprofloxacin and quercetin) were placed on different nutrient agar plates, swabbed with *Escherichia coli* MTTC 443 and *Staphylococcus aureus* MTTC 96 for antibacterial studies and incubated at 37°C. The GbH, loaded with 5-flucytosine, was placed on potato dextrose agar plates, swabbed with *Candida albicans* MTTC 183 for antifungal studies and incubated at 27°C. Controls were maintained by using the GbH without any drug.

**Agar overlay method**

The GbH, loaded with drugs, was used to study the effect of a microbial load on the GbH through the agar overlay method. The GbH and the drugs (mixed in DMSO) were in the ratio of 2:1. Later on, 10 mL of GbH-loaded ciprofloxacin and quercetin drugs were placed on a nutrient agar plate. A 0.7% soft agar (0.2-0.5% of working volume) was inoculated with *E. coli* MTTC 443 and *S. aureus* MTTC 96 and was poured on separate plates of the GbH for antibacterial studies, incubated at 37°C. Similarly, the GbH, loaded with 5-flucytosine, was placed on a potato dextrose agar plate, and soft agar with *C. albicans* MTTC 183 was overlaid on the GbH for antifungal studies, incubated at 27°C. Controls were maintained by using the GbH without any drug.

**Patch-agar method**

The GbH, loaded with drugs, was used to prove its suitability of the GbH as a dressing patch. The GbH and drugs (mixed in DMSO) were in the ratio of 2:1, and 1 cm^2^ of the GbH, with the same length of the inert medical wound, were placed on nutrient agar plates and potato dextrose agar plates to test their antibacterial and antifungal properties, respectively. Plain cotton and cotton loaded with drugs were placed on the inert medical wound and used as negative and positive controls, respectively. The test and controls were inoculated with a swab of *E. coli* MTTC 443 and *S. aureus* MTTC 96 for antibacterial studies, incubated at 37°C. Antifungal plates were inoculated with *C. albicans* MTTC 183 and incubated at 27°C. The test was conducted for three days, and on each successive day, a swap was taken from the inoculated area to test for bacterial and fungal growth on nutrient agar plates and potato dextrose agar plates for antibacterial and antifungal activities, respectively.

**Toxicity studies**

**Determination of GbH stabilisers and cell viability**

Cell lines: The first level of cytotoxicity biological assay to determine the stabiliser for GbH was done using L929 mouse fibroblast. All the resources and cell lines were provided in collaboration with the Division of Thrombosis Research, Sree Chitra Tirunal Institute for Medical Sciences and Technology, Trivandrum, India. The second level of cytotoxicity biological assay to Optimise the stabiliser ratio through direct contact of GbH was done using 3T6 mouse fibroblast. All the resources and cell lines were provided in collaboration with Microbiology Lab, CSIR-Central Leather Research Institute, Chennai, India. The third level of cytotoxicity biological assay to Optimise the stabiliser ratio through direct and leachate of GbH was done using HaCat cell line fibroblast. All the resources and cell lines were provided in collaboration with Jamora Lab, IFOM-inStem Joint Research Laboratory, National Centre for Biological Sciences, Bangalore, India

The *in vitro* cytotoxicity analysis was performed with L929 cell lines using a modified MTT assay to evaluate the biological protection of the GbH ^12^. Cells were grown in Dulbecco's modified eagle medium (DMEM) with 10% fetal bovine serum (FBS) in a full medium, using antibiotics for 48 h, followed by differentiation for 72 h in 96-well plates, at a final concentration of 1$\times$10^4^ cells per well. When the wells entered the confluent level, the medium was replaced with a fresh medium and the MTT assay was conducted on the GbH with various stabilisers, such as quercetin (QU), eugenol (EU), vitamin C (Vc) and sodium metaperiodate (SmP), at concentrations ranging from 1-20 mg. The stabilisers, along with the gelatin, were mixed with the GbH during the semi-polymerised stage. The polydopamine-to-stabiliser ratio in GbH was maintained between 1:0.5 and 1:5. The controls included the GbH, with and without polydopamine. The wells were filled with ~2.0 mg UV-sterilized gels and conducted in triplicates for a 24 h duration. After incubation, the medium and the GbH were gently removed and the MTT solution (5 mg/mL in PBS) was added to the plates and incubated at 37°C for 3 h. Later, the MTT solution was discarded and 100 μL of DMSO was added to dissolve the formazan crystals, and the absorbance level was measured at 570 nm using BIO-RAD iMark plate reader.

**Optimisation of stabiliser ratio through indirect contact of GbH**

In order to validate the polydopamine-to-SmP ratio, MTT cell viability assay was also performed using 3T6 cell lines. This experiment also aids in determining the GbH's indirect contact (leachate) toxicity. As per above mentioned MTT assay protocol the absorbance was measured at 570 nm on the Tecan Infinite M200 Pro, Bioscreen microplate reader. The ratio of polydopamine-to-SmP was maintained from 1:0.5 to 1:5, which was determined.

**Optimisation of stabiliser ratio through direct and leachate of GbH**

The results of the second MTT assay were further considered for the leachate toxicity assay. The final assessment of the GbH cytotoxicity was carried out by means of an MTT assay in compliance with Polish standards PN-EN ISO 10993-5:2009, using 3T6 and HaCat cell lines ^13^ and the absorbance was measured at 570 nm using Tecan Infinite M200 Pro, Bioscreen (for 3T6) and Synergy HT (for HaCat) microplate reader. The non-toxic concentration of the stabiliser was considered for the preparation of leachate extracts (100, 50 and 25 wt%) from the GbH and hydrogel. The cells were cultured in the 96-well plates, with the medium at the final concentration of 6$\times$10^3^ cells per well. As the cells reached the confluent state, the culture medium was replaced with (i) a fresh culture medium (positive control); (ii) the supernatant was collected from the incubated GbH (24 h contact; 0.1g GbH/mL) and (iii) a fresh culture medium with 30% DMSO (negative control) was tested.

**Brine shrimp lethality**

The *Artemia salina* eggs (Brine shrimp eggs) were hatched under ambient light and aeration as required for 24 h and upon the conformation of newly-hatched nauplii, the brine shrimp lethality assay (n = 10) was undertaken with a slight modification of the previously mentioned protocol ^14^. A 1 cm^2^ GbH piece (direct contact) test sample was analysed for brine shrimp lethality. The test solutions of 24 h GbH-leachate extracts (indirect contact) were tested in different ratios of 0.5:1 and 1:1 with the media and were also analysed for brine shrimp lethality. The experiment was performed in triplicates with appropriate controls.

**Zebrafish acute fish toxicity**

- *Danio rerio* (Zebrafish in 2:3 ratio, two male and three female, full-grown, healthy and adult stage)
- E3 medium composition: 34.8 g sodium chloride (NaCl), 1.6 g potassium chloride (KCl), 5.8 g calcium chloride (CaCl_2_·2H_2_O), 9.78 g magnesium chloride (MgCl_2_·6H_2_O)
- E3 medium preparation: Prepare a 60X stock, dissolve the salts in H_2_O to a final volume of 2 L, and adjust the pH to 7.2 with NaOH and autoclave. Dilute 16.5 mL of the 60X stock to 1 L (1X medium) and add 100 µL of 1% methylene blue (Sigma-Aldrich)

Full-grown, healthy adult zebrafish (2:3; two male and three female) were isolated in a breeding cage and were observed for eggs at the bottom of the fish tank. The presence of zebrafish eggs at the bottom of the tank confirmed the mating of fishes that were separated from the eggs. The eggs were collected from the bottom of the tank into a falcon tube with an E3 medium. Each egg was observed under a light microscope to confirm healthy eggs and separated (n = 10) for fish embryo acute toxicity (FET) with a slight modification of the previously mentioned protocol in accordance with the Organisation for Economic Co-operation and Development (OECD) guidelines for the testing of chemicals, section 2; Test No. 212: FET Test ^15^. Various apical observations such as coagulated embryos, lack of somite formation, non-detachment of the tail and lack of heartbeat were recorded for each tested embryo every 24 h until 96 h post-fertilisation (hpf) and the severity scaling of the toxicity of the GbH was recorded. Test solutions of 24 h GbH-leachate extracts were tested in different ratios of 0.5:1 and 1:1 with the media. The media for the FET assay was E3 and the 1 cm^2^ GbH piece test sample was also analysed for FET assays. The experiment was performed in triplicates with appropriate controls.

***In vivo* acute dermal toxicity studies**

**Animals and experimental plan**

The experiment was carried out at the Institute for Industrial Research and Toxicology, F-209, U.P.S.I.D.C., M.G. road, Ghaziabad-201302, India and the experiment was labelled as Project No.: 202112-25; Report No: IIRT/TOX/202112/ADT/0112; Date: 14-12-2021. All methods were carried out in accordance with guidelines and regulations of the Committee for the Purpose of Control and Supervision of Experiments on Animals (CPCSEA), New Delhi, India. The methods implemented in the current study are in accordance with ARRIVE Guidelines 2.0 ^16^. A protocol detailing the acute dermal toxicity studies, treatment groups and design of the experiment is mentioned in SI Materials and Methods. The protocol for the dermal toxicity study was for 14 days and animals were euthanised by isoflurane overdose using a small animal anaesthesia system.

The acute dermal toxicity study was carried out as per the OECD Guideline 402 ^17^. Healthy Wistar albino rats (10 males and ten nulliporous and non-pregnant females) weighing 200 to 250 g were taken and assigned into two groups depending on the dose level, Group-I: 2000 mg/kg body weight (Range finding study: 05 Male, 05 Female) and Group-II: 2000 mg/kg body weight (Confirmatory test: 05 Male, 05 Female).

**Acute dermal toxicity protocol**

The test animals were prepared 24 h prior to applying the test compound. The approximately 10% body surface area on the back of each animal was shaved by using electric clippers and care was taken to avoid abrasions during clipping. The test compound at the dose level of 2000 mg/kg body weight (limit test) in distilled water was applied uniformly over an exposed area. The test compound was in contact with the skin with an impervious dressing secured with adhesive tape. The animals were then housed individually in cages with a collar around the neck to avoid the ingestion of the test compound. After 24 h, the dressing was removed and the application site was cleaned with lukewarm water wiping the test compound.

**Clinical observation and mortality**

The treated animals were closely observed for clinical signs of intoxication for the first 4 h and thereafter for every 1 h interval for 24 h after dosing and twice a day for 14 days. All the rats were observed at least twice daily to record ill-health symptoms or behavioural changes. These observations included changes in skin and fur in the eyes and mucous membranes, respiratory, circulatory, central nervous and autonomous systems, somatomotor activity and behaviour changes to characterise erythema, hypersensitivity and oedema. Necropsy was carried out on all the animals that died during the study and surviving animals were sacrificed at termination. The LD50 value with fiducial limits at a 95% confidence level was calculated per standard protocol.

**Histopathological of dermal region**

A rat from each group was euthanised on day 14 after the wounding for histopathological examination. Samples of tissue (2x3 mm) placed in buffered formalin (10%), dehydrated by alcohol, have been excised and inserted in paraffin wax blocks. In assessing pathological modifications, thin pieces of tissue samples (5 μm) were stained with hematoxylin and eosin (H and E) ^17^. Test tissues from each group were preserved in 10% neutral buffered formalin and dehydrated in graded concentrations of ethanol, immersed in xylene and then embedded in paraffin. The sections of 4 µm thickness were cut and placed on the slide using commercial Baker’s mounting fluid. Paraffin wax was removed by warming the slide gently until the wax melted and then was washed with xylene. This was followed by washings with absolute alcohol and water to hydrate the sections and stained with haematoxylin and eosin described by Clayden (1971) ^18^. The hydrated sections were stained with haematoxylin for 15 min. The stained sections were washed with water and treated with a 1% acid alcohol mixture for 20 s. The acid alcohol mixture was washed off with water and sections were counterstained with a 1% aqueous solution of eosin for 2 min. After washing with water to remove excess eosin, the sections were dehydrated using absolute alcohol and then mounted using Canada balsam as a mounting agent. The slides were examined under an Olympus CX 41 microscopes for gross histopathological changes and neutrophil accumulation.

**Wound healing studies**

***In vitro* scratch wound healing studies**

The cell migration of the HaCat cell in the presence of the GbH and quercetin-loaded GbH was established using a standard scratch wound healing assay protocol with a slight modification of the media replacement method ^19^. The GbH was immersed in the cell culture medium for 24 h to obtain leachate, in compliance with Polish standards PN-EN ISO 10993-5:2009 and subjected to scratch wound healing assay ^20^. The cells were cultured in the 24-well plates with the medium at the final concentration of 1$\times$10^4^ cells per well and were seeded. As the cells reached the confluent state, the culture medium was replaced as follows: (i) a fresh culture medium and cell culture medium incubated 37°C for 24 h (positive control); (ii) leachate of the GbH in various concentrations of 25%, 50% and 100% (24 h contact, i.e., 0.1 g GbH/mL) and (iii) a fresh culture medium with 30% DMSO (negative control) was tested. Appropriate scratches were drawn using micropipette tips at an angle of roughly 90° to keep the scratch width confined. The wound area was photographed and examined using an inverted microscope (AXIOCAM 105 color) at 40x and 100x magnification.

***In vivo* second-degree burn wound healing studies**

**Animals and experimental plan**

Rats (either sex), weighing between 250-300 g, were procured from the Disease-Free Small Animal House Facility (DFSAH) of Lala Lajpat Rai University of Veterinary and Animal Sciences (LUVAS), Hisar, Haryana, India. All methods were carried out in accordance with guidelines and regulations of Committee for the Purpose of Control and Supervision of Experiments on Animals (CPCSEA), New Delhi, India. The methods implemented in the current study are in accordance with ARRIVE Guidelines 2.0 ^16^. The animals were quarantined and housed at the Central Animal House Facility (CPCSEA Registration no. 1753 Wistar /PO/E/S/14/CPCSEA) for acclimatisation for seven days before experimentation. The experimental animals were divided into seven groups (n = 6), and the protocol duration was 21 days. After 21 days the animals were euthanised by isoflurane overdose using small animal anaesthesia system.

**Second-degree burn wound protocol**

The induction of second-degree burn injuries was carried out by standard protocol ^21^ ^22^. The scalding wound model was used to study the rate of wound contraction, period of epithelialisation, biochemical-, connective tissue-, immunological-parameters and histopathological analysis. The burn wounds were inflicted on the dorsal thoracic region 1- 1.5 cm away from the vertebral column and 5 cm away from the ear. Carefully, animals were restrained in the rat holder, and 2 cm of the area on the dorsal where the burn wound was to be inflicted was carefully shaved to expose the skin. Hot water (80°C) was poured over the shaved area for 7 s. The heat exposure caused a uniform second-degree burn on the skin. The experimental animals were divided into seven groups (n = 6) (note: the animals assigned/ approved for the control group was n = 10) and the duration of the protocol was 21 days. The groups and the design of the experiment were assigned as follows;

| **Group** | | **No. of animals** |
| --- | --- | --- |
| Group I | Normal/ sham group | n = 6 |
| Group II | Burn injury/ control group (Control) | n = 10 |
| Group III | Control cream-base treated group (Cream) | n = 6 |
| Group IV | 1% quercetin cream-base treated group (QC) | n = 6 |
| Group V | Control GbH-base treated group (Hydrogel) | n = 6 |
| Group VI | 1% quercetin-GbH treated group (QH) | n = 6 |
| Group VII | 1% silver sulfadiazine cream-treated group (SS). | n = 6 |

The design of the experiment for each group;

| **Groups** | **Treatment** |
| --- | --- |
| I | - Not subjected to burn - No treatment - Serve as normal or sham |
| II | - Subjected to burn - No treatment |
| III | - Subjected to burn - External application cream formulation |
| IV | - Subjected to burn - External application 1% quercetin cream formulation |
| V | - Subjected to burn - External application GbH formulation |
| VI | - Subjected to burn - External application 1% quercetin GbH formulation |
| VII | - Subjected to burn - External application silver sulfadiazine cream formulation |

The prevention of spinal shock was undertaken, and the animals were immediately resuscitated with an intraperitoneal injection of ringer lactate solution (2 mL/100 g body weight). Following the injection of ringer lactate solution, the formulations (with and without any medicament) were applied to the injured area as per the group distribution as mentioned earlier for 21 days between 10:00 and 11:00 AM using a sterile gauge to cover the entire burned area uniformly. Burn wound healing was assessed by measuring the percentage of wound contraction during 21 days and observed for the above parameters.

**Pathological examination**

**Measurement of wound contraction**

The wound margin was traced on a transparent paper after wound creation. The area of the wound was measured and plotted for day 4, 7, 14 and 21 ^23^. The wound healing area was expressed in percentage by using the formula,

Percentage wound contraction = $\frac{initial wound size - specific day wound size}{initial wound size} \times100$

**Study of epithelialisation period**

The number of days required for the eschar to fall off from the wound surface without leaving a raw wound behind was noted ^23^.

**Measurement of tensile strength**

The tensile strength of a wound healed skin represents the quality of healed wound. The skin specimen is stretched at a particular force and the repaired tissue resisting the tension indicted the quality of repaired tissue. The animals were anaesthetised and sacrificed by decapitation, skin tissue samples were collected and processed according to approved standard protocol ^23^. The quality of the burn-healed skin when compared to a normal group was evaluated using a wound stretching machine, EFG500E, EFGE digital force gauge on day 21.

**Biochemical evaluation of burn wound**

**Sample preparation: Skin tissue homogenate**

After decapitation on day 21, a full thickness of the healed and natural skin (1 cm^2^) was cautiously removed. The tissue was weighed and then homogenised with a glass homogeniser at 4°C in 1x PBS (tissue weight (g): PBS (mL) Volume = 1:9) and centrifugation was followed at 10,000g at 4°C for 30 min. The samples used for histological observation were deposited in 10% neutral buffered formalin ^23^.

The biochemical parameters include malondialdehyde (MDA) ^24^, glutathione (GSH) ^25^ and catalase (CAT) ^26^ levels of the homogenised skin sample according to standard protocols, which were analysed using UV/Visible double beam spectrophotometer, Shimadzu and expressed in nmoles MDA/mL, μ mol/g tissue and µ moles of hydrogen peroxide utilised/mg/tissue/min, respectively.

**Estimation of malondialdehyde**

- 1 mM Tetramethoxy Propane (TMP) standard solution: 1 mM solution of 1, 1, 3, 3-teteramethoxy propane was prepare by stepwise dilution of 1 M solution (0.82/5mL, v/v) and stored in 4 ᵒC. Further, a working standard of 20 nM/mL from 1 mM solution was diluted to yield a final concentration of 16, 12, 10, 8, 4, 2, 1 nM/mL, and a standard curve was plotted for the estimation of total MDA.

A mixture of supernatant and Tris-HCl (each 1 mL) was incubated at 37 °C for 2 h. Later to this 1 mL of 10% trichloroacetic acid reagent (TCA) was added and centrifuged at 10,000g for 10 min. The supernatant obtained was added to 0.375% w/v thiobarbituric acid reagent (TBA) (each 2 mL), and the tubes were kept in boiling water for ten minutes. After cooling, 1 mL of distilled water was added, and absorbance was measured at 532 nm using a Shimadzu UV/Visible double beam spectrophotometer. The standard curve for the estimation of total MDA was plotted as mentioned above with different dilutions of TMP. The extent of lipid peroxidation was expressed using the formula,

$$nmoles MDA/ml=\frac{\text{ 6.41 × Absorbance sample × Total volume}}{\text{volume of sample}}$$

**Estimation of glutathione**

- Ellman’s reagent: 19.8 mg of 5,5′-dithiobis(2-nitrobenzoic acid) (DTNB) in 100 mL of 0.1% sodium citrate

The assay of GSH with DTNB was performed by following a standard Ellman’s method, 1959. Briefly**,**  1.5mL potassium phosphate buffer and 0.5 mL supernatant were mixed properly. Further to the mixture 0.5 mL Ellman’s reagent was added and thoroughly shaken, thereafter incubated at 30 °C for 5 min. The standard curve was plotted in the same above method in a range of 0.2-1 mM using standard GSH. The absorbance was recorded at 412 nm using Shimadzu UV/Visible double beam spectrophotometer, and GSH blank was also prepared in which GSH was omitted.

**Estimation of catalase**

- Dichromate/acetic acid: This reagent is prepared by mixing 50 mL of a 5% aqueous solution of potassium dichromate with 150 mL of glacial acetic acid

A mixture of 0.1 mL of the homogenate, 1 mL of phosphate buffer and 0.5 mL of hydrogen peroxide was allowed to react for 60 s. The reaction was arrested by the addition of 2 mL of dichromate acetic acid reagent. Then the tubes were heated in a boiling water bath for 10 min. The green colour developed was read at 570 nm in Shimadzu UV/Visible double beam spectrophotometer. The standard curve was plotted in the same above method in a range of 10-160 mM using standard 0.1 mL of hydrogen peroxide (H_2_O_2_).

**Connective tissue evaluation of burn wound**

**Sample preparation: Hydrolysate**

The wet tissue (Approximately 250 mg) was dried at 50°C for 24 h in glass stoppered test tubes. Later, to the 40 mg of the dried granulation tissue, 1 mL of 6 N HCl was added and exposed to a boiling water bath for 24 h. After cooling of the hydrolysate, excess acid was neutralised by 10 N NaOH using phenolphthalein. The neutral hydrolysate was diluted to a concentration of 20 mg/mL with distilled water, which was further used to estimate hydroxyproline (HXP) and hexosamine (HXA) ^27^. The standard curve was prepared using the proper substrate and the HXP and HXA content of granulated tissues were expressed in μg/mL of tissue.

**Estimation of hydroxyproline**

A volume of 0.3 mL of hydrolysate, 2.5 N NaOH, 0.01M CuSO_4_, and 6% H_2_O_2_ was mixed well, shaken vigorously, and placed in a water bath at 80°C for 15 min. In the same tube, after they were cooled down, 0.6 mL of freshly prepared 5% of paradimethyl amino-benzaldehyde in n-Propanol and 1.2 mL of 3 N sulphuric acid (H_2_SO_4_) were added and added incubated in a hot water bath at 75°C for 15 min and then cooled. The intensity of the resultant color was measured at 540 nm against the blank using Shimadzu UV/Visible double beam spectrophotometer. The standard curve of hydroxyproline content in the hydrolysate was prepared with standard 4-Hydroxy-L-proline, from 75 to 900 μg/0.3 mL using a 3 mg/mL working solution.

**Estimation of hexosamine**

A volume of 0.05 mL of hydrolysate made up to 0.5 mL with distilled water. The 0.5 mL of acetylacetone reagent in the diluted test sample was added and incubated in a boiling water bath for 20 min. After cooling, a volume of 1.5 mL of 95% alcohol and 0.5 mL of Ehrlich’s reagent was added and incubated at RT for 30 min. The mixed solution was kept for 30 min to complete the reaction. The intensity of the resultant color was measured at 530 nm against the blank using Shimadzu UV/Visible double beam spectrophotometer. The standard curve of hexosamine content in the hydrolysate was prepared with standard D-(+)-glucosamine hydrochloride.

**Estimation of NF­**-**κB levels**

The NF-κB in the skin tissue was estimated with Rat NF-κB (Nuclear factor kappa B) enzyme-linked immunoassay (ELISA) Kit, based on the Sandwich-ELISA principle. (Biolab Technology Laboratory, Shanghai Korean Biotech Co., Ltd)

**Sample preparation: Skin tissue homogenate**

After decapitation on day 21, a full thickness of the healed and natural skin (1 cm^2^) was cautiously removed. The tissue was weighed and then homogenised with a glass homogeniser at 4°C in 1x PBS (tissue weight (g): PBS (mL) Volume = 1:9) and centrifugation was followed at 10,000g at 4°C for 30 min.

**Estimation of NF-κB**

Strips required for the assay were determined based on the samples and strips were inserted into the frame. A volume of 50 μL of the standard solution was added to 40 μL of the sample as well as to the standards provided by the kit, followed by the addition of 10 μL anti-nfκb antibody and later 50 μL streptavidin-HRP. Contents were mixed well, sealed with a sealer and placed in the incubator for 60 min at 37°C. After incubation, the sealer was removed, and the plate was washed five times with wash buffer by soaking each well with 0.35 mL wash buffer for 30 s to 1 min. After washing 50 μL substrate solution A and 50 μL substrate solution B were added to each well. The plate was once again covered with a new sealer and incubated at 37°C in the dark for 10 min. The reaction was stopped by 50 μL stop solution, read at 450 nm using a microplate reader (Alere AM 2100), and expressed in ng/mL of tissue.

**Molecular docking of quercetin and anti-inflammatory targets**

**Preparation of ligand and targets**

The crystallographic 3D protein structures of the p50 (PDB ID: 1SVC), IκBα (PDB ID: 1NFI), NEMO/IKK (PDB ID: 3BRV), NF-κB homodimer (PDB ID: 1SVC) and TNF receptor (PDB ID: 2E7A) were retrieved from the RCSB protein data bank (<https://www.rcsb.org>). The available 3D ligand structure of quercetin was derived from the PubChem library (https://pubchem.ncbi.nlm.nih.gov/).

**Molecular docking using Autodock**

Molecular docking was carried out using Autodock 4.2 software and tools such as Open Babel, CASTp, Discovery studio visualisation (2D) and PyMol (cartoon) for conversion of file formats, predicting the active site and data visualisation, respectively ^28^. The target molecules underwent the removal of the water molecule, the addition of polar hydrogens, the confirmation of torsion angles and the addition of Kollman charges. Furthermore, the localisation of the binding position, in order to generate the grid map, was followed by the Lamarckian genetic algorithm protocol used to initiate molecular docking.

**Histopathological estimations**

After day 21, one rat from each group was euthanised for histopathological examination and burn wound-healed tissues Samples of (2x3 mm) were cut and placed in buffered formalin (10%) and dehydrated in graded concentrations of ethanol, immersed in xylene and then embedded in paraffin. The sections of 4 µm thickness were cut and placed on the slide using commercial Baker’s mounting fluid. Paraffin wax was removed by warming the slide gently until the wax melted and then was washed with xylene. Later, it was followed by washings with absolute alcohol and water to hydrate the sections and stained with haematoxylin and eosin described by Clayden (1971) ^18^. The hydrated sections were stained with haematoxylin for 15 min. The stained sections were washed with water and treated with a 1% acid alcohol mixture for 20 s. The acid alcohol mixture was washed off with water and sections were counterstained with a 1% aqueous solution of eosin for 2 min. After washing with water to remove excess eosin, the sections were dehydrated using absolute alcohol and then mounted using Canada balsam as a mounting agent. The slides were examined under Olympus CX 41 microscopes for gross histopathological changes and neutrophil accumulation.

**Statistical analysis**

**For retrieval trails and Biochemical parameters**

The results were analysed using one-way Anova followed by Tukey’s *post-hoc* analysis with *p* ≤ 0.05 considered significant for all values on GraphPad Prism 8.4.3.686.

**Reference**

1. Hassan, A., Niazi, M. B. K., Hussain, A., Farrukh, S. & Ahmad, T. Development of Anti-bacterial PVA/Starch Based Hydrogel Membrane for Wound Dressing. *J. Polym. Environ.* (2018) doi:10.1007/s10924-017-0944-2.

2. Ju, K. Y., Lee, Y., Lee, S., Park, S. B. & Lee, J. K. Bioinspired polymerisation of dopamine to generate melanin-like nanoparticles having an excellent free-radical-scavenging property. *Biomacromolecules* (2011) doi:10.1021/bm101281b.

3. OU, A. & BO, I. Chitosan hydrogels and their glutaraldehyde-crosslinked counterparts as potential drug release and tissue engineering systems - synthesis, characterisation, swelling kinetics and mechanism. *J. Phys. Chem. Biophys.* (2017) doi:10.4172/2161-0398.1000256.

4. Benedict, S. R. A reagent for the detection of reducing sugars. 1908. *J. Biol. Chem.* (2002) doi:10.1016/s0021-9258(18)91645-5.

5. Wu, F., Pang, Y. & Liu, J. Swelling-strengthening hydrogels by embedding with deformable nanobarriers. *Nat. Commun.* (2020) doi:10.1038/s41467-020-18308-9.

6. ASTM. Standard test methods for water vapor transmission of materials E96/E96M. *Annu. B. ASTM Stand.* (2013).

7. Roy, N., Saha, N., Kitano, T., Vitkova, E. & Saha, P. Effectiveness of polymer sheet layer to protect hydrogel dressings. *Prog. Colloid Polym. Sci.* (2010) doi:10.1007/978-3-642-19038-4_22.

8. Hago, E. E. & Li, X. Interpenetrating polymer network hydrogels based on gelatin and PVA by biocompatible approaches: Synthesis and characterisation. *Adv. Mater. Sci. Eng.* (2013) doi:10.1155/2013/328763.

9. Pal, K., Banthia, A. K. & Majumdar, D. K. Polyvinyl alcohol-gelatin patches of salicylic acid: Preparation, characterisation and drug release studies. *J. Biomater. Appl.* (2006) doi:10.1177/0885328206056312.

10. Dash, S., Murthy, P. N., Nath, L. & Chowdhury, P. Kinetic modeling on drug release from controlled drug delivery systems. *Acta Poloniae Pharmaceutica - Drug Research* (2010).

11. Preem, L. *et al.* Monitoring of antimicrobial drug chloramphenicol release from electrospun nano-and microfiber mats using UV imaging and bacterial bioreporters. *Pharmaceutics* (2019) doi:10.3390/pharmaceutics11090487.

12. Owonubi, S. J., Mukwevho, E., Aderibigbe, B. A., Revaprasadu, N. & Sadiku, E. R. Cytotoxicity and in vitro evaluation of whey protein-based hydrogels for diabetes mellitus treatment. *Int. J. Ind. Chem.* (2019) doi:10.1007/s40090-019-0185-4.

13. Boido, M. *et al.* Chitosan-based hydrogel to support the paracrine activity of mesenchymal stem cells in spinal cord injury treatment. *Sci. Rep.* (2019) doi:10.1038/s41598-019-42848-w.

14. Banti, C. & Hadjikakou, S. Evaluation of toxicity with brine shrimp Assay. *BIO-PROTOCOL* (2021) doi:10.21769/bioprotoc.3895.

15. Tomić, S. L., Nikodinović-Runić, J., Vukomanović, M., Babić, M. M. & Vuković, J. S. Novel hydrogel scaffolds based on alginate, gelatin, 2-hydroxyethyl methacrylate, and hydroxyapatite. *Polymers (Basel).* (2021) doi:10.3390/polym13060932.

16. du Sert, N. P. *et al.* The arrive guidelines 2.0: Updated guidelines for reporting animal research. *PLoS Biol.* **18**, (2020).

17. Chakrabarti, S. *et al.* Safety profile of silver sulfadiazine-bFGF-loaded hydrogel for partial thickness burn wounds. *Cutan. Ocul. Toxicol.* (2018) doi:10.1080/15569527.2018.1442843.

18. Richards, O. W. & Clayden, E. C. Practical Section Cutting and Staining. *Trans. Am. Microsc. Soc.* (1973) doi:10.2307/3224944.

19. Zhang, X. *et al.* Stimulation of wound healing using bioinspired hydrogels with basic fibroblast growth factor (bFGF). *Int. J. Nanomedicine* (2018) doi:10.2147/IJN.S168998.

20. ISO/EN10993-5. ISO 10993-5 Biological evaluation of medical devices - Part 5: Tests for cytotoxicity: in vitro methods. *Int. Stand. ISO* (2009).

21. Guo, H.-F., Ali, R. M., Hamid, R. A., Zaini, A. A. & Khaza’ai, H. A new model for studying deep partial-thickness burns in rats. *Int. J. Burns Trauma* (2017).

22. Bhatia, N., Kaur, G., Soni, V., Kataria, J. & Dhawan, R. K. Evaluation of the wound healing potential of isoquercetin-based cream on scald burn injury in rats. *Burn. Trauma* (2016) doi:10.1186/s41038-016-0032-1.

23. Dwivedi, D., Dwivedi, M., Malviya, S. & Singh, V. Evaluation of wound healing, anti-microbial and antioxidant potential of Pongamia pinnata in wistar rats. *J. Tradit. Complement. Med.* (2017) doi:10.1016/j.jtcme.2015.12.002.

24. Buege, J. A. & Aust, S. D. Microsomal Lipid Peroxidation. *Methods Enzymol.* (1978) doi:10.1016/S0076-6879(78)52032-6.

25. Beutler, E. *Glutathione in Red Cell Metabolism : A manual of Biochemical methods*. *J. Lab. Clin. Med* (1975).

26. Aebi, H. E. *Catalase in Methods of Enzymatic Analyses*. *Methods of Enzymatic Analyses* (1983).

27. Murthy, S. *et al.* Evaluation of in vivo wound healing activity of Bacopa monniera on different wound model in rats. *Biomed Res. Int.* (2013) doi:10.1155/2013/972028.

28. Morris, G. M. *et al.* AutoDock4 and AutoDockTools4: Automated docking with selective receptor flexibility. *J. Comput. Chem.* (2009).
